# Whole-brain functional hypoconnectivity as an endophenotype of autism in adolescents

**DOI:** 10.1016/j.nicl.2015.07.015

**Published:** 2015-08-07

**Authors:** R.L. Moseley, R.J.F. Ypma, R.J. Holt, D. Floris, L.R. Chura, M.D. Spencer, S. Baron-Cohen, J. Suckling, E. Bullmore, M. Rubinov

**Affiliations:** a Department of Psychiatry, Brain Mapping Unit, University of Cambridge, Cambridge, UK; b University of Cambridge, Hughes Hall, Cambridge, UK; c Autism Research Centre, Department of Psychiatry, University of Cambridge, Cambridge, UK; d Cambridge Lifespan Asperger Syndrome Service (CLASS) Clinic, Cambridgeshire and Peterborough National Health Service Foundation Trust, Cambridge, UK; e Department of Experimental Psychology, Behavioural and Clinical Neuroscience Institute, University of Cambridge, Cambridge, UK; f Cambridgeshire & Peterborough National Health Service Foundation Trust, Cambridge, UK; g ImmunoPsychiatry, Alternative Discovery & Development, GlaxoSmithKline, Stevenage, UK; h Churchill College, University of Cambridge, Cambridge, UK

## Abstract

Endophenotypes are heritable and quantifiable markers that may assist in the identification of the complex genetic underpinnings of psychiatric conditions. Here we examined global hypoconnectivity as an endophenotype of autism spectrum conditions (ASCs). We studied well-matched groups of adolescent males with autism, genetically-related siblings of individuals with autism, and typically-developing control participants. We parcellated the brain into 258 regions and used complex-network analysis to detect a robust hypoconnectivity endophenotype in our participant group. We observed that whole-brain functional connectivity was highest in controls, intermediate in siblings, and lowest in ASC, in task and rest conditions. We identified additional, local endophenotype effects in specific networks including the visual processing and default mode networks. Our analyses are the first to show that whole-brain functional hypoconnectivity is an endophenotype of autism in adolescence, and may thus underlie the heritable similarities seen in adolescents with ASC and their relatives.

## Highlights

• Adolescents with autism and genetic relatives show weaker functional connectivity.
• This appears at a whole-brain level in several task contexts.
• Connectivity in autism and in siblings is much more variable than in controls.
• Local endophenotypes also appear in default mode network and visual network.
• Hypoconnectivity is a feature of the broader and the diagnosable autism spectrum in adolescents.

## 1 Introduction

Autism spectrum conditions (ASCs) are common pervasive neurodevelopmental conditions which typically present in early childhood and manifest with characteristic impairments in communication and social relationships, alongside unusually repetitive behaviours and restricted interests. Numerous studies have shown ASC to be highly heritable (Ronald and Hoekstra, 2011; Berg and Geschwind, 2012; Colvert et al., 2015), with genetic heritability estimated at 80% (Lichtenstein et al., 2010); one recent estimate suggests that siblings of people with autism are 7 times more likely to be diagnosed with an ASC than are members of the general population with no genetic relationship to an autistic proband (Grønborg et al., 2013). The complex polygenic interactions underlying ASC give rise to a continuous spectrum of subclinical and clinically diagnosed presentations (Baron-Cohen et al., 2001a; Hoekstra et al., 2007).

The combination of high genetic heritability and heterogeneous presentation supports the search for endophenotypes of autism. Endophenotypes are the heritable and quantifiable – neurophysiological, biochemical, endocrinological, neuroanatomical, cognitive or behavioural – markers of psychiatric conditions (Gottesman and Gould, 2003). Endophenotypes are found in patients and their asymptomatic relatives, and thus simplify the search for causes of complex psychiatric conditions by identifying genetically mediated and quantifiable traits that bind together diverse clinical presentations (Kalueff et al., 2015). Here we search for endophenotypes in a matched sample of males with ASC, unaffected siblings of people with ASC, and typically developing controls.

A broader autistic phenotype (BAP) (Losh et al., 2009) has been identified at the behavioural level, with genetic relatives of autistic probands exhibiting more autistic traits than the general population (Constantino et al., 2010; Wheelwright et al., 2010) and more aloof, anxious, and rigid personality styles (Piven et al., 1997; Hurley et al., 2007). Whilst not meeting diagnostic criteria for ASC, they also display abnormalities and impairments resembling the core symptomatology of ASC in the domains of communication, social reciprocity and behavioural rigidity (Piven et al., 1997; Pickles et al., 2000; Losh et al., 2009), along with deficits in elements of executive function like planning, set-shifting and verbal fluency (Wong et al., 2006; Nydén et al., 2011).

In contrast to the behavioural BAP, there is limited evidence for a corresponding broader neural phenotype of autism. Previous reports have shown that ASC-like cognitive profiles in relatives are associated with atypical brain activation in localised brain regions (Dawson et al., 2005; Baron-Cohen et al., 2006; Spencer et al., 2011, 2012a, 2012b; Holt et al., 2014). Such studies produce spatial activation maps but do not describe interactions between brain regions. In contrast, functional connectivity analyses study distributed networks of correlated activity between brain regions (Fox and Raichle, 2007; Van Dijk et al., 2010). ASCs are now increasingly understood to present with system-wide differences in neural information processing (Minshew and Goldstein, 1998; Belmonte et al., 2004a, 2004b; Welchew et al., 2005; Geschwind and Levitt, 2007; Kana et al., 2011; Vissers et al., 2012; Uddin et al., 2013; Di Martino et al., 2014), and are conceptualised as “nonfocal, systemic… distributed neural systems disorder[s]” (Minshew and Goldstein, 1998), rather than disorders of focal brain regions. The search for autism endophenotypes in brain connectivity is a fledgling field. Early reports show altered functional connectivity in genetically high-risk infants (Orekhova et al., 2014; Righi et al., 2014; Keehn et al., 2015), and reduced white matter maturity in siblings of individuals with ASC (Lisiecka et al., 2015), but no study has previously examined a functional-connectivity endophenotype in adolescence.

The current evidence for whole-brain connectivity differences between typically developing individuals and those with ASC is inconsistent (Barttfeld et al., 2011, 2012; Müller et al., 2011; Rudie et al., 2012; Vissers et al., 2012; Ray et al., 2014; Tyszka et al., 2014). This inconsistency may partly arise from heterogeneity of participants; both age (Sowell et al., 2003; Westlye et al., 2010; Lebel and Beaulieu, 2011) and sex (Lai et al., 2012, 2013), for instance, are known to affect neuropathology in ASC, whilst intelligence is known to modulate several aspects of neurobiology, potentially including information transfer and connectivity (Luders et al., 2009; Neubauer and Fink, 2009). Studies investigating psychological or neurobiological processes in non-matched age, gender and IQ participants risk masking true differences or reporting false positives (Mottron, 2004). Other reasons for inconsistency may include the difficulty of comparing studies which explore connectivity in distinct task states. Such disparities represent an important anomaly to the notion of ASCs as "developmental disconnection syndromes" (Geschwind and Levitt, 2007).

Here, we addressed the above limitations by testing whole-brain hypoconnectivity as an endophenotype of autism in a gender-, age-, and IQ-matched sample of adolescents, at rest and across a range of tasks, thus simultaneously maximising sample homogeneity and task diversity. We employed complex network analysis (Bullmore and Sporns, 2009; Rubinov and Sporns, 2010) to characterise functional-MRI connectivity networks. This approach conceptualises neuroimaging data as a complex system of nodes (brain regions) and connections (interregional functional associations) and has successfully revealed organisational abnormalities in many severe psychiatric conditions but is a fledgling field in autism research (Menon, 2011; Rubinov and Bullmore, 2013). We have focused our analysis on description of whole-brain connectivity, but have also reported observed differences in local connectivity for completeness and for comparison with previous work.

The strengths of our study for identification of whole-brain autism endophenotypes lie in the unique combination of a well matched sample of participants, the presence of unaffected siblings, the presence of multiple functional tasks and rest, and in whole-brain complex network analysis.

## 2 Materials and methods

### 2.1 Participants

We analysed a matched group of 14 adolescent males with diagnosed ASC, 14 unaffected male siblings of individuals with ASC (henceforth “siblings”), and 14 typically-developing controls with no family history of ASC (Table 1), matched from a larger pool of 68 male and 60 female participants (Spencer et al., 2011; Spencer et al., 2012a, 2012b; Floris et al., 2013; Holt et al., 2014). All of the data and measures analysed in this study were collected in these previous works, the protocols for which were approved by the Cambridgeshire 1 Research Ethics Committee (National Health Service).

As mentioned above, matching is crucial in autism research, given that individual differences in age, gender and IQ between participants can confound results. Our 14-participant groups were selected using an automated and unbiased matching algorithm (‘MATCH’) which minimised pairwise distances between participants in the normalised feature space (van Casteren and Davis, 2007). The numbers of participants in our study represent the maximal possible number with absent between-group differences in age, gender, and full-scale IQ (Wechsler, 1999). In addition, matching for full-scale IQ alone can leave discrepancies between ASC and typically-developing individuals in the subscales of verbal and nonverbal IQ (Jarrold and Brock, 2004), and our groups were in fact matched on all three measures. Diagnostic status of the ASC group was confirmed with the Autism Diagnostic Observational Schedule-Generic (ADOS-G) (Lord et al., 2000) and the Autism Diagnostic Interview-Revised (ADI-R) (Le Couteur et al., 2003). Siblings and controls scored significantly lower in number of autistic traits as measured on the Autism-Spectrum Quotient (AQ) (Baron-Cohen et al., 2001b), and on the Social Communication Questionnaire (SCQ) (Rutter et al., 2003), they scored below the cut-off that differentiates them from people with ASC. Siblings did not differ statistically from controls in these measures.

The majority of siblings in our study (11/14) were not related to the participants with ASC: this largely avoids the potential confound introduced by shared environmental factors in the sibling and ASC groups (Cannon and Keller, 2006). None of the participants were currently or had previously taken psychotropic medication, though it was later revealed that one sibling had a diagnosis of resolved childhood epilepsy not initially reported during participant screening.

### 2.2 Procedure

The data analysed here were collected by Spencer and colleagues, and the full details of the experimental tasks and the rationale for choosing them were published previously (Spencer et al., 2011, 2012a, 2012b; Floris et al., 2013; Holt et al., 2014). Having completed the psychometric tests reported above, participants performed three short cognitive tasks (counterbalanced for order) during functional MRI recordings. Behavioural performance and group differences in brain activity during each task have been published in the above reports.

One task (henceforth “Eyes task”) was adapted from the “Reading the Mind in the Eyes” task (Baron-Cohen et al., 2001a), and simultaneously tested “theory of mind” and emotion recognition. Participants were presented with a pair of eyes and asked to either choose one of two mental state words to describe the expression of the eyes (mental state condition), or to decide whether the eyes were male or female (gender judgement condition); see Holt et al. (2014) for details.

Another task involved detection of small component shapes within a complicated visual pattern, the so-called Embedded Figures task (henceforth “Figures task”: see Spencer et al., 2012b, for details) which has been popularly used in autism research and often demonstrates superior performance in people on the spectrum (Shah and Frith, 1983; Jolliffe and Baron-Cohen, 1997; Bölte and Poustka, 2006).

Another task (henceforth “Ekman task”) used the Ekman (1975) pictures of facial affect, requiring participants to make judgements of gender on faces that were either happy, fearful or neutral and thus scrutinising the effects of emotion on cognitive processing (Spencer et al., 2011).

Recordings were also taken of the cohort during ‘resting state’, a 7-minute period where participants were simply asked to close their eyes and think of nothing.

### 2.3 Imaging analysis

Functional and structural MRI scans were acquired on a Siemens Tim Trio 3-T system (Siemens Healthcare, Erlangen, Germany) at the MRC Cognition and Brain Sciences Unit (CBSU) in Cambridge, UK. In a sequence lasting 4 min and 32 s, MPRAGE structural images were acquired with the following parameters: repetition time (TR) = 2250 ms, echo time (TE) = 2.98 ms, inversion time (TI) = 900 ms, flip angle 9°, voxel size 1 × 1 × 1 mm. Echoplanar (EPI) images during the functional tasks were acquired in a descending interleaved pattern with the following parameters: TR = 2000 ms, TE = 30 ms, flip angle 78°, voxel size 3 × 3 × 3 mm, field of view = 192 × 192 mm, 64 × 64 acquisition matrix. 32 slices were acquired with a slice thickness of 3 mm and an inter-slice distance of 0.75 mm.

The data preprocessing pipeline employed MRIcron (Rorden et al., 2007), AFNI (Cox, 1996) and FSL (Jenkinson et al., 2012). The first five scans of each functional EPI series were discarded to ensure signal equilibration. Following skull-stripping, brain segmentation and non-linear registration to MNI space, anatomical images were co-registered with functional scans, which had been realigned and slice-time corrected. We extracted motion parameters using AFNI 3dvolreg and identified the mean contribution of cerebrospinal fluid (CSF) and white matter to the signal by creating trimmed (partial volume estimates >0.99) binary masks of both. We then regressed out these confounds, their derivatives, and quadratic terms (a total of 32 regressors, as described in Satterthwaite et al., 2013; Patel et al., 2014), but not global signal (Gotts et al., 2013). We used AFNI's 3dBandpass command to despike each participant's time-series, apply an 0.01 Hz high-pass filter and an optional 0.1 Hz low-pass filter (see below for a discussion), regress the confounds and smooth the time-series with an 8-mm FWHM Gaussian kernel.

We parcellated the images using a parcellation scheme of 264 8 mm regions of interest (ROIs), split into 14 functional networks (Power et al., 2011): see Supplementary Materials, 1. Six ROIs were located outside of the brain in several participants, and were thus discarded. Using MATLAB, we calculated functional connectivity between these remaining 258 nodes using the Pearson correlation coefficient. We removed weak and potentially spurious correlations by preserving only the strongest 20% of connection weights for each participant, and analysing the resulting weighted matrices (Rubinov et al., 2009; van Wijk et al., 2010). This proportional thresholding represents the most popular approach in complex network analysis, and through normalising individual subject weight, emphasises differences in network topology, rather than differences in total connectivity. In this context, proportional thresholding can reduce and thus if anything underestimate any present between-group differences in total connectivity.

In order to observe whether deviations in functional connectivity are task-dependent, we included cognitive tasks and resting state data in our analysis. Previous investigations of functional connectivity diverge in methods depending on whether they concern resting state or task-based data. So-called “intrinsic” functional connectivity reflects the low-frequency, spontaneous fluctuations in connectivity that appear during rest (Fox et al., 2007; Van Dijk et al., 2010). Autism researchers also frequently analysed functional connectivity during cognitive tasks, an approach henceforth described as “task-evoked” which often does not involve low-pass filtering, in order to emphasise task effects. Since previous studies have shown that the use of a low-pass filter substantially modulates findings in functional autism connectivity datasets (Jones et al., 2010; Nair et al., 2014), we completed analyses with and without low-pass filtering. Our main analyses included a low-pass filter, and are reported throughout the results section; additional analyses without low-pass filtering are reported and compared in Results 3.4.

### 2.4 Correction of movement

Movement is a critical issue in functional connectivity as it can create artefacts of hypoconnectivity (Power et al., 2012, 2014, 2015; Satterthwaite et al., 2012, 2013; van Dijk et al., 2012). The correction of movement artefact is a difficult problem without a clear consensus (Power et al., 2015); it is worth noting that whilst censoring of time-points has been prominently advocated as a solution (Power et al., 2013, 2014), this procedure has advantages and disadvantages, and does not represent an accepted gold standard (Beall and Lowe, 2014; Power et al., 2015).

We performed several quality control checks to ensure that group differences did not reflect motion artefacts. Six location parameters were extracted from the scans of each participant for each slice during the scan time-series. We computed the mean framewise displacement (following Jenkinson et al., 2002; Power et al., 2012), as the sum of the absolute values of the derivatives of the translational and rotational realignment estimates (after converting rotational estimates to displacement at 50 mm radius), and averaged it to define mean motion for each participant over the whole scan. We also identified the maximum spike of movement (i.e. the largest difference in the location parameters between slices). We found no gross of movement of participants on visual inspection and consequent statistical analysis revealed that the groups did not differ significantly in mean motion (F(2, 41) = .925, p = .405) or in the number of movement spikes (F(2, 40) = 2.036, p = .145).

**Fig. 1:**
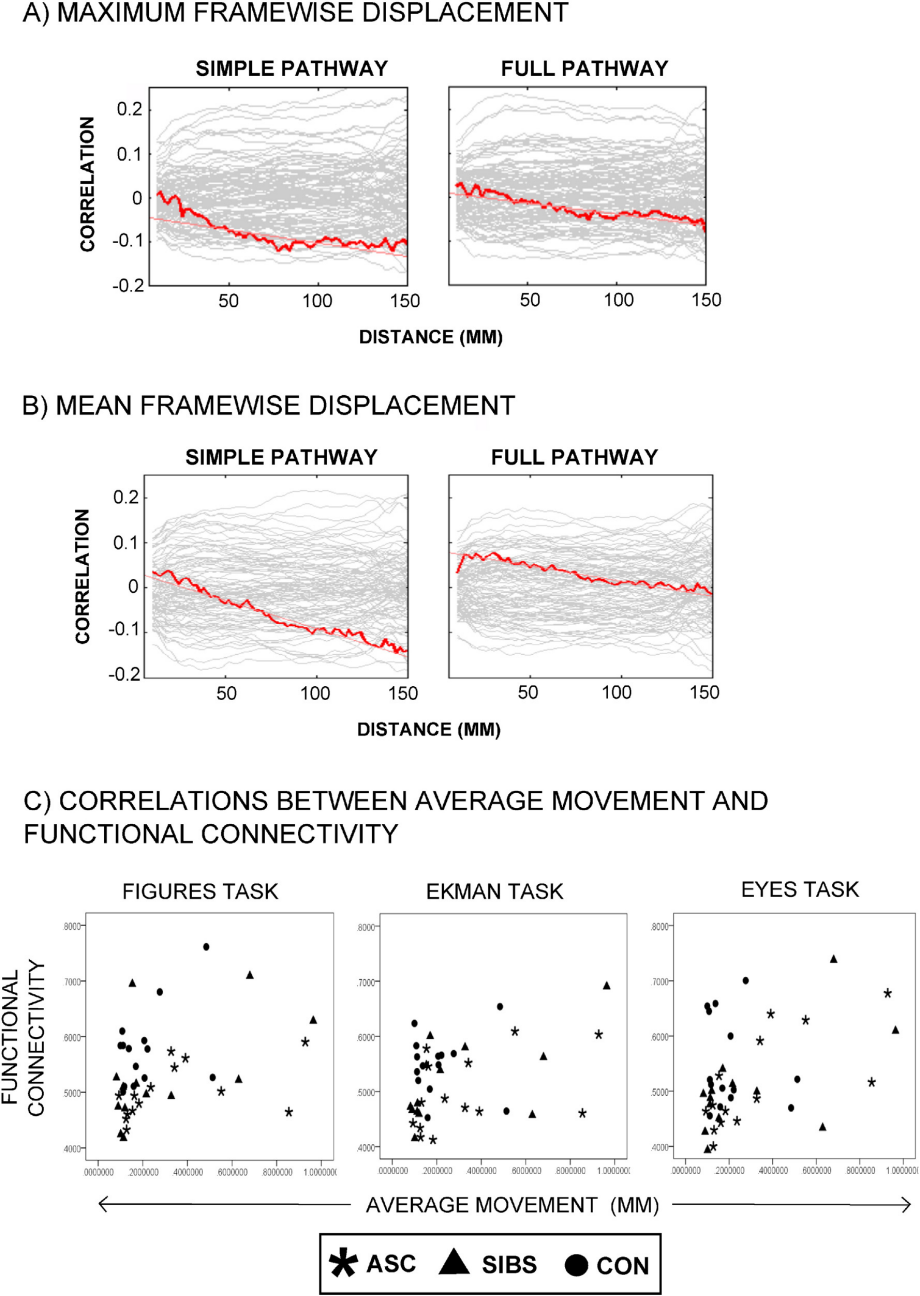
Moving average of correlation between maximum framewise displacement (A) or mean framewise displacement (B) and functional connectivity against distance between nodes. This is shown in each case for the simple pipeline without motion correction and for our full pipeline. The bold red lines reflects values from actual data, whilst straight red lines are fitted linear functions: grey lines are obtained by permuting movement values for participants. (C) Correlations between average movement and functional connectivity in each task. As can be seen, most participants are clustered together with low average movement. The few outliers who moved most belonged to the ASC (stars) and sibling (triangle) groups and appear to be consistent across each task, but these participants in fact tend to show higher functional connectivity.

To further investigate a possible influence of motion on our results, we computed, for each pair of nodes, the correlation between functional connectivity and maximum framewise displacement. Fig. 1, Part A shows the moving average of these correlations as a function of Euclidean distance between nodes, for a “null-hypothesis” pipeline with only CSF and white-matter but no motion correction, and for our full preprocessing pipeline. In the absence of a movement artefact we would expect the correlations to be around zero. In the presence of an artefact we would expect higher correlations for short-distance node pairs and lower correlations for long-distance node pairs (Power et al., 2013; Patel et al., 2014). To assess the magnitude of the correlations, we performed the same computations after permuting the framewise displacement values for the participants 100 times, thus generating the distribution of values to be expected when no relationship between motion and functional connectivity exists (the grey lines of Fig. 1A and 1B). We then tested, for both pipelines, if the overall mean correlation or the distance dependence is significantly different from those observed in the permutations. The overall mean correlation was not significantly different from the null distribution for both pipelines (p > 0.1), but we found that the slope of a straight line fitted through the correlation values at a steeper angle for the simple pipeline (slope: −0.6/m, p value < 0.01), where it was almost flat for our full pipeline (slope: −0.5/m, p value 0.1). This is consistent with a possibly artefactual relationship between movement and distance dependence in the data, which is largely corrected for by our pipeline. The use of maximum framewise displacement is a more stringent test for motion corruption that has been omitted in previous studies (which typically used mean framewise displacement). When we examined the relationship between motion and mean framewise displacement (Fig. 1B), the effect size of the distance-dependent artefact was negligible.

Finally, we examined the correlation between mean movement and maximum movement (spike) parameters and global functional connectivity in each task. Global functional connectivity did not correlate with movement spikes, but did correlate with mean movement in the Figure (r = .339, p = .030), Ekman (r = .348, p = .026) and Eyes (r = .412, p = .007) tasks. Positive correlations between functional connectivity and average movement (see Fig. 1C) reflected that participants who moved more tended to have higher functional connectivity. This bolsters the interpretation of genuine hypoconnectivity in the ASC group rather than artefactual hypoconnectivity resulting from greater movement.

Important recent work suggests that that movement may also represent a biological, in addition to artefactual, correlate of dysconnectivity (Zeng et al., 2014) In this context, we did not regress group-average motion estimates in our analysis to avoid removal of important biological effects which are correlated with, but do not arise as a result of, varying levels of motion.

### 2.5 Statistical analysis of endophenotypes in functional connectivity

The central tenets of the endophenotype concept (Gottesman and Gould, 2003) suggest that if disrupted connectivity were present as an *endophenotype* of autism, we would predict differences between autistic participants and controls, and between siblings and controls. Significant difference between affected individuals and their genetic relatives are of interest but not strictly necessary for identification of endophenotypes, so we do not include them. For each variable in the forthcoming analysis, we therefore employed analysis of variance (ANOVA) to identify group differences and followed this with t-tests comparing siblings and controls and comparing participants with ASC and controls. In all cases, we used the IBM Statistical Package for the Social Sciences (SPSS). Given the novel and exploratory nature of this investigation, we did not correct for multiple comparisons.

#### We analysed global network organisation with four measures:

*Whole-brain functional connectivity*: We examined connection strengths in weighted matrices (pairwise correlations between 258 brain regions), searching for group differences when collapsing all tasks. Many studies of functional connectivity have taken an a priori approach focusing on certain ROIs. Whilst this is certainly a valid approach based on an abundance of previous literature, results from these analyses have been somewhat inconsistent (Müller et al., 2011). In this context, we focused on a potentially more robust data-driven whole-brain analysis.

*Clustering coefficient* (*C*): *C* quantifies the number of connections between a node's nearest neighbours and reflects the density of edges (connections) in a node's immediate neighbourhood (Rubinov and Sporns, 2010: see paper for details). To ensure that *C* in each participant differed from that which would be expected by chance and degree distribution alone, for each participant we divided the value of *C* by the mean value of *C* obtained from an ensemble of 100 random networks with the same size, density, degree and strength distribution of the participant template on which they were based (Rubinov and Sporns, 2011).

*Global efficiency* (*E*): *E* is defined as the average inverse shortest path length – the minimal number of edges (or ‘steps’) – between all pairs of nodes. The greater the path length of a network, the less efficient it is (Bullmore and Sporns, 2012) (see Rubinov and Sporns, 2010, for details). We normalised *E* as above.

*Node disruption index*: We examined the extent to which autistic and sibling participants differed from controls in node characteristics, using the measure of node disruption index (NDI: originally termed “hub disruption index”) (Achard et al., 2012). First, the total weight of connections (strength) was calculated for each node, and averaged over the control group. For each participant, these average nodal strengths were then subtracted from individual nodal strengths. The NDI is the slope of these differences against the average values over the control group, and represents the similarity of nodal properties to the average of nodal properties of the typical participant (see Supplementary Materials 2 and 3 for details and additional analyses).

*Local measures*: We analysed local differences in functional connectivity by examining the location of the most highly-connected nodes (“hubs”), defined as the 20% of nodes with highest total connection weight. The distribution of these hubs was explored in 9 of the 14 functional networks, namely the cerebellar, cingulo-opercular and frontoparietal task control, default mode, dorsal and ventral attention, salience, subcortical and visual networks. We excluded three networks irrelevant to the nature of our tasks (the auditory network and two sensorimotor networks), and two functionally imprecise networks (see Supplementary Material 1, for details). Having identified the network identity of hubs in each group, we searched for differences in the distribution of hubs between ASC, sibling and control participants by tallying, in each participant, the number of hubs in each of network. As before, ANOVAs were first conducted followed by t-test comparisons of ASC vs. controls and siblings vs. controls. We ensured the robustness of these results with additional analyses, including an alternative method of hub selection and an analysis of interregional and intranetwork connections (see Supplementary Material 4).

## 3 Results

### 3.1 Global differences: functional connectivity

**Fig. 2:**
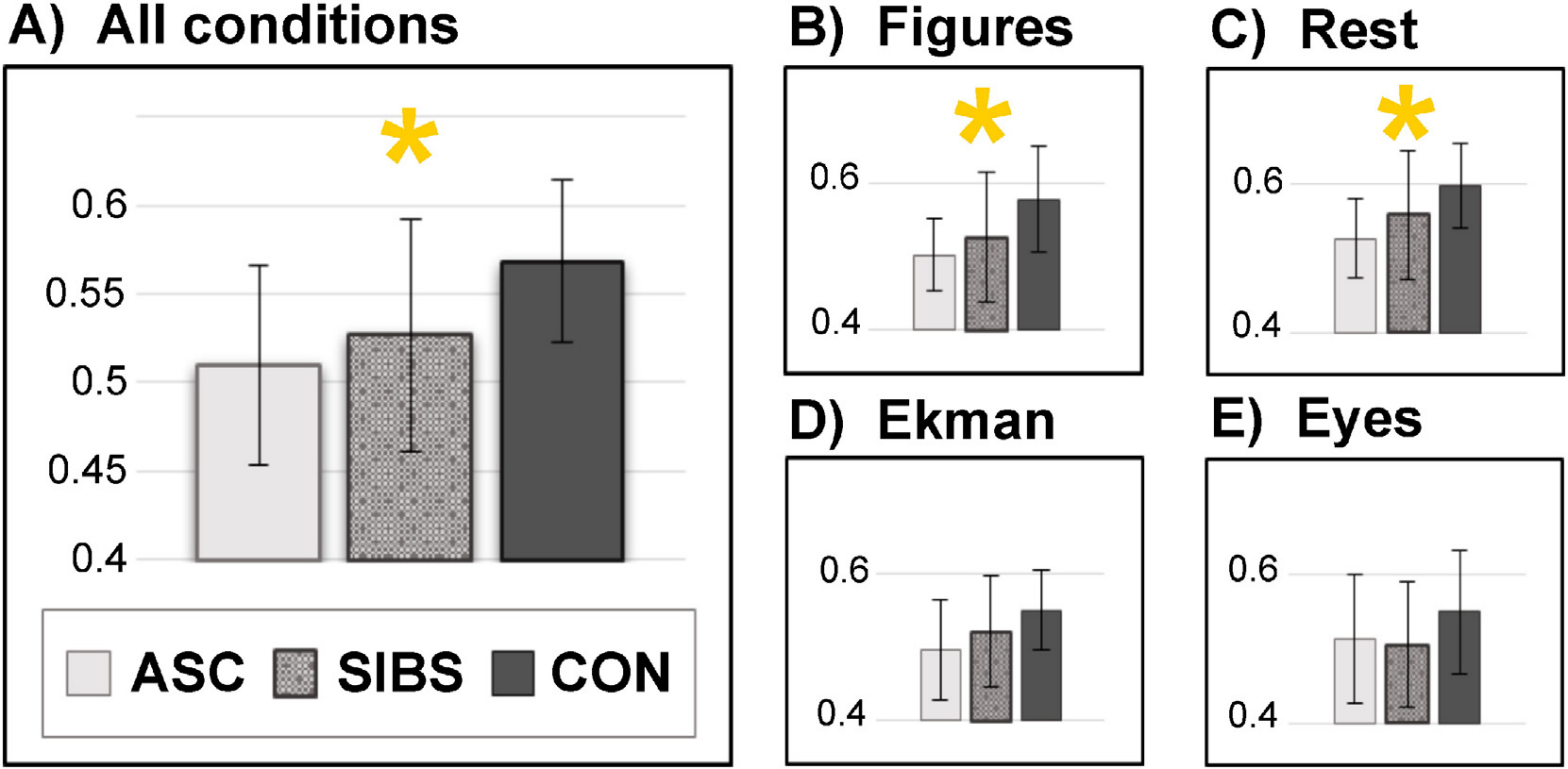
Average connection weights (correlation coefficients) computed from weighted matrices for each group during each task condition. Error bars represent standard deviation. Asterisks reflect significant (p < .05) group differences in ANOVAs: as can be seen, these emerged for all four conditions together and during the Figures task and resting state alone. The group difference was non-significant in the Ekman task.

In initial analysis of average connection weights between 258 brain nodes, we observed a significant effect of group with the inclusion of all four task conditions (*F*[1, 39] = 4.082, p = .025), reflecting differences in group connectivity averaged across all tasks. Fig. 2A illustrates that the control group showed the strongest global connectivity and the ASC group the weakest, with siblings intermediate. One-way ANOVAs of each task condition individually showed significant group differences in connection weights in the Figures task (*F*[2, 41] = 4.003, p = .026: Fig. 2B) and during resting-state (*F*[2, 41] = 4.221, p = .022: Fig. 2C), and a non-significant trend in the same direction (p = .118: Fig. 2D) in the Ekman task. There was no significant trend effect in the Eyes task (Fig. 2E); on post-hoc consideration, this could reflect issues around the nature of this task, which conflated mentalising conditions and gender-judgement conditions such as to be a non-specific task of ‘active cognitive processing’. For this reason, we focus on the more easily interpretable task states for the remainder of the paper, but report analysis of the Eyes task in Supplementary Materials (5) for completeness and transparency.

Strongly significant correlations (each with a p-value lower than .01) were seen between connectivity in each of the conditions (see Supplementary Materials, 6). This reflected the fact that individuals showed differences in connectivity across the board rather than in any one task, which explains the main effect of Group when all tasks were collapsed (Fig. 2A). Individuals with lower connectivity tended to be those with ASC and, to a lesser extent, siblings.

**Fig. 3:**
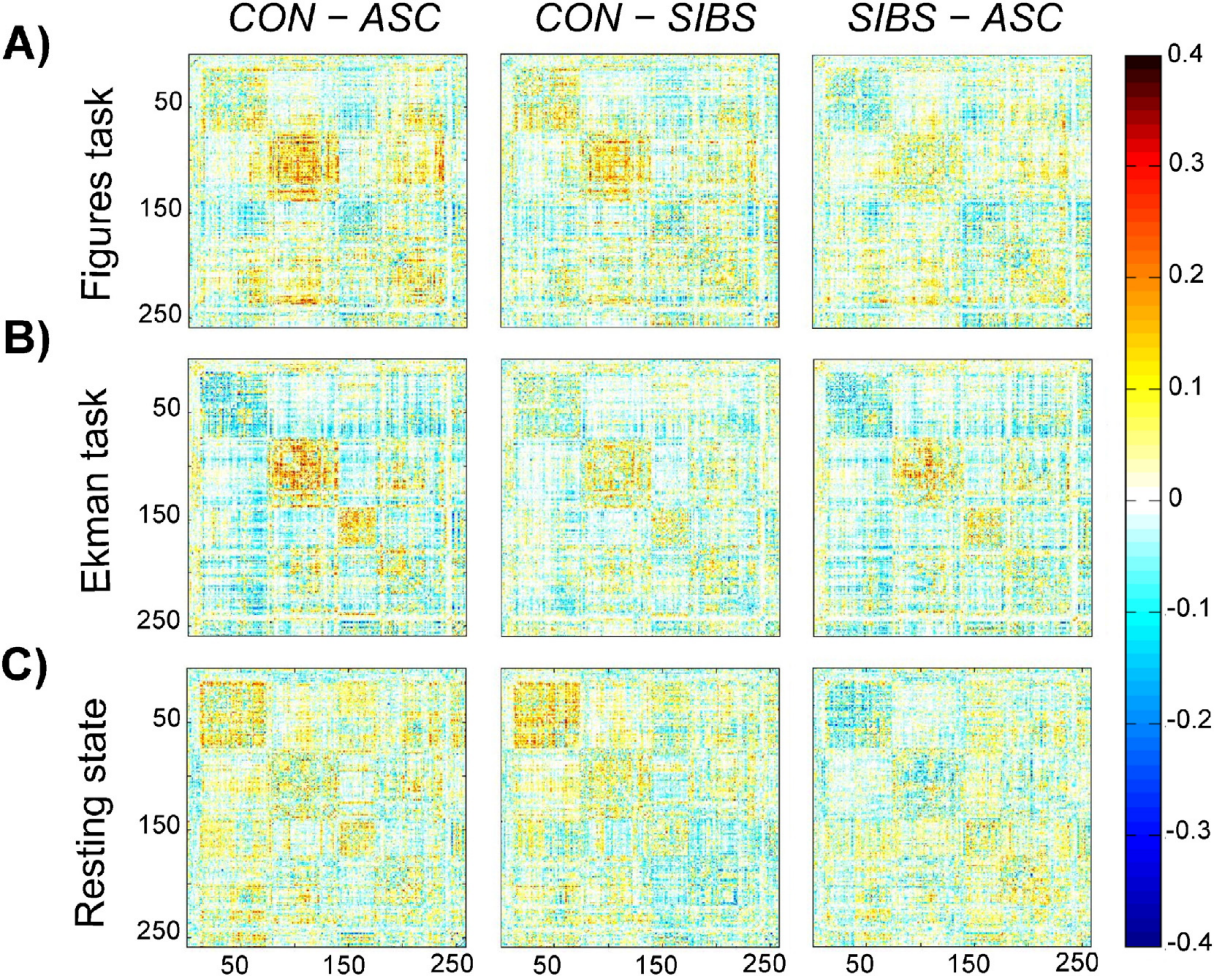
Difference matrices produced by subtracting the connection weights of one group from another. The 258 × 258 matrices reflect the edges (connections) between 258 brain nodes. White voxels reflect connections where groups do not differ in connection weights. Red voxels represent connections which are stronger in the first group than the second (controls > ASC; controls > siblings; siblings > ASC). Blue voxels represent connections which are stronger in the second group than the first (ASC > controls; siblings > controls; ASC > siblings).

The difference between control and ASC participants was significant in the Figure (*t*[26] = 3.291, p = .003) and Ekman tasks (*t*[26] = 2.270, p = .032) and during rest (*t*[26] = 3.427, p = .002), and ASC participants showed significantly lower whole-brain connectivity than controls when all tasks were collapsed (*t*[26] = 2.748, p = .011). T-tests found no significant differences between siblings and control participants in any task condition (p > .3 for each tasks; although p = .072 across all tasks, see Fig. 2, Panel A). Panels A and B suggest that siblings are more similar to the ASC group in the strength of functional connectivity (see Fig. 3 for an alternative representation).

Significant endophenotype effects appeared in the non-normalised clustering coefficient (*C*) and global efficiency (*E*) in the Figures task, the Ekman task and during rest, reflecting differences between control, sibling and ASC participants in global network organisation. These were not significant when normalised by reference null models (Supplementary Materials, 7).

### 3.2 Global differences: network organisation

**Fig. 4:**
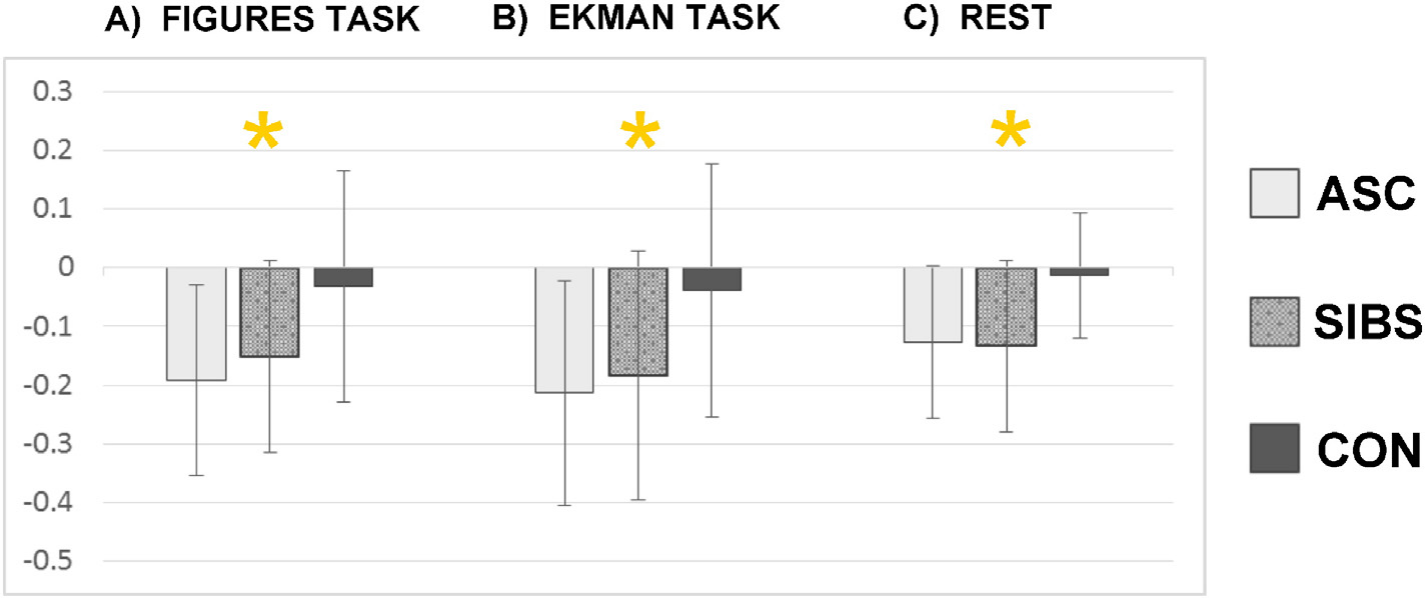
Mean node disruption index (NDI) for each group in each task with standard deviation in error bars. In each task, the mean NDI of control participants close to zero reflected that with little variance, each tended to resemble the group average in nodal strength. Mean NDIs further away from zero reflected deviance from typical node structure in the autistic and sibling groups. Asterisks represent significant (p < .05) differences between groups in ANOVA.

The similarity between autistic and sibling participants was further evident in node disruption index (NDI). Scores closest to zero reflect similarity to nodal strength in the typical brain (Fig. 4), and the three groups differed significantly in the Figures task (*F*[2, 41] = 5.322, p = .009), the Ekman task (*F*[2, 41] = 4.422, p = .019), and during rest (*F*[2, 41] = 5.452, p = .008). Siblings and control participants differed significantly in the Figures task (*t*[26] = 2.226, p = .035) and during rest (*t*[26] = 2.491, p = .019); the other comparison relevant for endophenotypes, that of ASC vs. controls, was also significant in the Figures task (*t*[26] = 3.354, p = .002), the Ekman task (*t*[26] = 3.061, p = .005) and during rest (*t*[26] = 3.458, p = .002).

This measure reflects homogeneity within the control group, given that NDI scores were significantly closer to zero in individual control participants than in individuals in the ASC or the sibling group. Control participants would, however, be naturally expected to resemble the template computed from their group average strength. Therefore, to examine the homogeneity of the ASC and sibling groups themselves, we computed deviance from average nodal strength when the ASC or sibling group were used as an average. Whilst individual controls were close to an average control template of node strength, siblings and participants with ASC were no closer to their group's average nodal strength than were the other groups: no group differences were seen in any task, reflecting greater heterogeneity in ASC and sibling groups than in control participants. We report this fully in Supplementary Materials (3).

### 3.3 Local changes in network topography

We examined the presence of localised changes in connection differences by considering the distribution of hub nodes in individual brain networks, defined by our 258-node parcellation scheme.

**Fig. 5:**
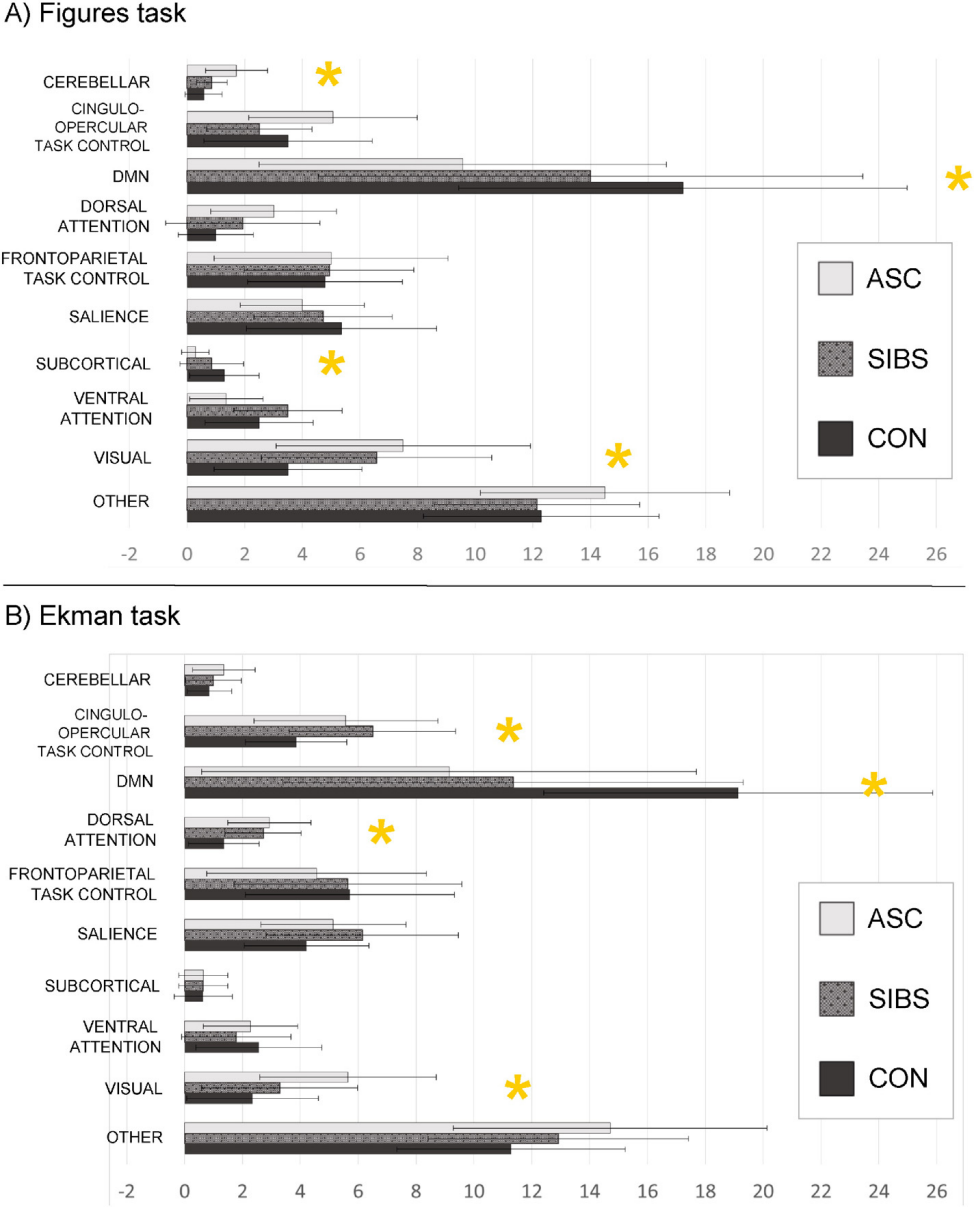
Average distribution of the 20% highest-strength hubs over 9 functional networks in the Figure (A) and Ekman tasks (B), with error bars reflecting standard deviation. Asterisks represent significant group differences (p < .05) in ANOVA.

The topography of hubs differed substantially in the Figure and Ekman tasks. Two patterns emerged during the Figures task (Fig. 5A). Group differences characterised by low number of hubs in controls and high number of hubs in ASC appeared in the cerebellar network (*F*[2, 41] = 8.048, p = .001) and the visual network (*F*[2, 41] = 4.379, p = .019), with siblings intermediate in both. In direct comparisons, siblings differed statistically from controls only in the visual network (*t*[26] = 2.421, p = .023); ASC and control participants also differed significantly in the visual network (*t*[26] = 2.931, p = .007), reflecting a true endophenotype effect. ASC participants also differed from controls with more hubs in the cerebellar network (*t*[26] = 3.432, p = .002), but the siblings did not differ significantly from the controls.

A reversed endophenotype effect, with autistic participants showing the fewest hubs and controls the most, was evident in the subcortical network (*F*[2, 41] = 3.672, p = .035) and was marginally non-significant in the default mode network (DMN) (*F*[2, 41] = 3.095, p = .057). T-tests between siblings and controls were not significant in either case, though controls and ASC participants differed significantly in the number of hubs in the subcortical (*t*[26] = 2.895, p = .008) and default mode (*t*[26] = 2.721, p = .011) networks.

Finally, non-endophenotype (non-linear) effects were observed in the ventral attention network (ASC participants showed the fewest hubs and siblings the most, *F* [2, 41 = 5.594, p = .007); and in the cingulo-opercular task control network ASC participants showed the most hubs and siblings the fewest, *F*[2, 41] = 3.450, p = .042). No t-test comparisons were significant.

We confirmed all of these group differences using an alternative method of hub definition and further tests of local network connection weights. In this case, the group difference in the DMN, which was previously marginally non-significant, became significant and showed a strong endophenotype effect in intra-network and interregional connection weights (Supplementary Materials, 8).

Trends that were non-significant in the Figures task became significant during the Ekman task (Fig. 5B): autistic participants showed the fewest hubs and controls the most in the DMN (*F*[2, 41] = 6.381, p = .004). In contrast, control participants had the fewest hubs and ASC participants the most in the dorsal attention network (*F*[2, 41] = 5.746, p = .006). In both cases, siblings were intermediate but significantly different from controls in both the DMN (*t*[26] = 2.796, p = .010) and the dorsal attention network (*t*[26] = 2.823, p = .009): autistic participants also differed from controls in the number of hubs in the DMN (*t*[26] = 3.441, p = .002) and the dorsal attention network (*t*[26] = 3.121, p = .004). The reverse trend, with the greatest number of nodes in the ASC group and the fewest in the control group, was seen in the visual network with a group difference (*F*[2, 41] = 5.530, p = .008). T-tests did not however find this difference to be significant between siblings and controls.

Finally, a non-endophenotype effect was seen in the cingulo-opercular task control network, where siblings showed the greatest number of high-strength nodes and controls the fewest (*F*[2, 41] = 3.522, p = .039).

These differences remained significant with an alternative method of hub definition and in an analysis of interregional and intra-network connectivity (Supplementary Materials, 9).

### 3.4 The effect of low-pass filtering

The use of a low-pass filter has been shown to substantially influence results in autism neuroimaging (Jones et al., 2010; Nair et al., 2014). To check the robustness of our findings, we re-analysed our data without a low-pass filter. As a whole, our results remained consistent between these two preprocessing strategies, with no trend reversals and most, but not all, tests remaining significant. A full comparison of these two preprocessing strategies is in Supplementary Materials (10), and the main changes are as follows. In our analysis of correlation coefficients reflecting whole brain connectivity, t-tests showed that the comparison of connectivity between controls and ASC participants in the Ekman task became non-significant (*t*[26] = 1.790, p = .085); in contrast, a new significant difference now emerged between controls and siblings in resting state (*t*[26] = 2.257, p = .033). This, along with the difference between controls and ASC in resting state, would implicate hypoconnectivity during rest as a particular endophenotype in accordance with the criteria we adopted (see Materials and methods section, Section 2.4). In our analysis of node disruption index (NDI), the same trends remained but the group effects in the Figures task (*F*[2, 41] = 2.496, p = .095) and the Ekman task (*F*[2, 41] = 2.493, p = .096) dropped below significance, as did the contrast between controls and ASC participants (*t*[6] = 1.645, p = .112) and controls and siblings (*t*[26] = 1.975, p = .059) in the Figures task. Whilst this endophenotype did not therefore remain significant, NDI remained significantly different and an endophenotype effect remained in resting state. In the Figures task, group differences in the subcortical network and cingulo-opercular task control network became non-significant, but previously marginal group differences in the DMN (p = .057) became significant (*F*[2, 41] = 4.207, p = .022). In t-tests, the difference between controls and ASC participants in the subcortical network became marginally non-significant (*t*[26] = 1.963, p = .060), as did the difference between siblings and controls in visual network (*t*[26] = 1.960, p = .061). All local differences in the Ekman task remained significant.

## 4 Discussion

We explored whole-brain and local connectivity endophenotypes in a well-matched cohort of participants with ASC, unaffected siblings, and typically developing controls, and observed a robust whole-brain connectivity endophenotype effect in the Embedded Figures task, the Ekman task, and resting state. This was confirmed by several strands of analysis and with differences in preprocessing (see Supplementary Materials, 10). Primarily, we observed reduced correlation between brain regions across all tasks (and across some tasks individually): connection weights between regions were weakest in ASC, intermediate in siblings, and strongest in controls. Further analyses of strength similarity to typical brain connectivity, using the node disruption index (NDI), confirmed that both autistic and sibling participants deviated significantly from the average density of node connections as shown in the group of typically-developing adolescent boys — an effect which was present in all four conditions. Autistic and sibling participants did not differ significantly from each other in any task and were more heterogeneous, lacking the more similar node structure that made individual control participants close to their group mean in nodal strength. This heterogeneity or idiosyncrasy in the autistic brain extends the recent findings of Hahamy et al. (2015) by showing the genetic heritability underlying such idiosyncrasy.

Brain endophenotypes in the relatives of people with autism have been reported previously (Dawson et al., 2005; Baron-Cohen et al., 2006), including in studies which have analysed less stringently matched supersets of the current participant cohort (Spencer et al., 2011, 2012a, 2012b; Holt et al., 2014). These previous investigations reported localised changes in haemodynamic response (blood-oxygen level dependent: BOLD) to stimuli in task conditions. Analysis of functional connectivity endophenotypes in adolescent autism is, to the best of our knowledge, novel. Differences in functional connectivity may explain previously reported abnormalities in localised BOLD signal. For instance, abnormal functional connectivity may make it difficult to regulate and reduce brain activity (Spencer et al., 2012a), and may additionally underlie regional hypoactivity associated with autistic symptoms (Spencer et al., 2011, 2012b).

Functional hypoconnectivity might be interpreted as consistent, at a theoretical level, with the well-established weak central coherence account (Happé and Frith, 2006). Weak central coherence describes the tendency of people with ASC, their relatives (Briskman et al., 2001) and people with autistic traits (Best et al., 2008) to process data in a piecemeal fashion, biased towards local processing and often failing to process things in a global manner or to see the ‘bigger picture’. Just and colleagues (Just et al., 2012) link this and other autistic features to a disruption in the integrated activity of distributed brain regions underlying complex cognitive tasks. Of course, support for this theory rests on the assumption that the observed functional connectivity directly underlies integration and cognitive function*,* and the *behavioural correlate* of hypoconnectivity is far from transparent. For instance, despite the robust observed functional-connectivity effect, we did not observe a behavioural difference between siblings and controls in the Autism-Spectrum Quotient (AQ) or the Social Communication Questionnaire (SCQ). This may be due to the small size of our sample: previous studies demonstrating the heritability of the AQ have contained hundreds if not thousands of participants (Hoekstra et al., 2007; Ruzich et al., 2015). It is possible that behavioural tasks require more statistical power to detect an effect, whereas functional connectivity differences are more easily detected.

The existence of a whole-brain endophenotype in our dataset supports previous reports for heritability of functional connectivity (Posthuma et al., 2005; Smit et al., 2008; Schutte et al., 2013). Although where possible we avoided related ASC and sibling participants, it is impossible to completely disentangle genetic and environmental influences on the endophenotype, given that our unaffected siblings did live with their own autistic siblings. Gerdts et al. (2013), however, convincingly show a genetic continuum in the endophenotype with a comparison of families with one autistic child (simplex families) and families with multiple autistic children (multiplex families): the latter exhibit more restricted interests and repetitive behaviour and are less social, less likely to smile and to make eye-contact than participants from simplex families. Our analysis provides further biological evidence of inherited autistic features in the brain and may highlight genes involved in neural transmission as loci of interest.

### 4.1 Localisation of brain differences

The major focus of our analysis was on whole brain measures of connectivity. Analysis of hub topography revealed, however, that certain networks may be particularly compromised in siblings as well as individuals with autism. We mention several of these in light of their theoretical relationship to ASC.

During the Figures task, the cerebellar and visual networks had the greatest number of hubs in ASC and the fewest hubs in control participants. This appeared as an endophenotype effect in our primary analysis, although it lost significance when the low-pass filter was removed. The trend stayed consistent, however, and so this result may still be of interest, particularly for its consistency with previous research in autism. A greater number of high-strength nodes present in visual systems could be theoretically consistent with the strengths that both people with ASC and genetic relations exhibit in piecemeal processing (Baron-Cohen and Hammer, 1997; Bölte and Poustka, 2006). It is consistent, too, with previous reports of temporo-occipital and occipital hyperconnectivity reported in children and adolescents with ASC (Keehn et al., 2013; Keown et al., 2013). There has been suggestion that people with ASC show weaker connections between anterior and posterior brain regions (Just et al., 2004, 2007; Koshino et al., 2005; Kana et al., 2009; Damarla et al., 2010). We confirmed this difference in some of our confirmatory tests (see Supplementary Materials, 8). A more rigorous, focal investigation of connectivity in and involving the visual network in relatives could be a target for future research.

The most consistent effect in both the Ekman and the Figures task was found in the default mode network (DMN), where controls showed the greatest number, and autistics the fewest number, of high-strength hubs. This group effect was marginally non-significant with a low-pass filter (p = .057), but became significant without a low-pass filter (p = .022). Closer scrutiny of this finding (see Supplementary Materials, 9 and 10) confirmed that connectivity within the DMN and between the DMN and the rest of the brain was significantly weaker in autism and significantly weaker in siblings than matched controls, in both of these tasks. Components of the DMN decrease their activity during overt cognitive processing (Gusnard and Raichle, 2001; Raichle et al., 2001) and increase activity in ‘mind-wandering’ states (Greicius et al., 2003) and during tasks involving aspects of social cognition and mentalising (Schilbach et al., 2008; Lombardo et al., 2010; Mars et al., 2012). This does not appear to be the case in individuals with ASC (Kennedy et al., 2006). Consistent with their archetypal impairments in social cognition, functional hypoconnectivity of the DMN is a consistent finding in ASC (Kennedy and Courchesne, 2008; Assaf et al., 2010; Paakki et al., 2010; Weng et al., 2010; von dem Hagen et al., 2013; although see Lynch et al., 2013 and Redcay et al., 2013 for a divergent viewpoint). Our finding of DMN hypoconnectivity thus supports the notion that differences in DMN connectivity constitute a local endophenotype of autism in adolescence.

As we clustered individual regions together in functional networks, there may be additional local differences in connectivity (for e.g., see Keehn et al., 2013; Keown et al., 2013; Fishman et al., 2014) which were not detected by our analysis. We and others (Just et al., 2012) draw a tentative link between local connectivity of the visual system and visual processing strengths of ASC and genetic relatives, but pockets of hyperconnectivity in the brain may be as detrimental as hypoconnectivity to behaviour and function. This is evidenced in the relationship between hyperconnectivity and symptom severity or behavioural impairment (Mostofsky et al., 2007; Redcay et al., 2013; Supekar et al., 2013; Fishman et al., 2014). The mechanisms through which hyperconnectivity might result in functional impairment have been well discussed by several authors (Belmonte et al., 2004a; Courchesne and Pierce, 2005; Happé and Frith, 2006; Markram and Markram, 2010).

### 4.2 Strengths, limitations and future directions

Many previous reports of atypical connectivity in ASC have been localised to specific regions, and were potentially sensitive to methodological decisions (Jones et al., 2010; Nair et al., 2014); the multitude of differential approaches have made studies difficult to reproduce (Müller et al., 2011). Our finding of a whole-brain endophenotype in adolescents addresses previous questions about the whole-brain nature of autism connectivity abnormalities (Barttfeld et al., 2011, 2012; Rudie et al., 2013; Ray et al., 2014; Tyszka et al., 2014) and is backed up by other approaches such as ICA which have also reported whole-brain ASC hypoconnectivity (Mueller et al., 2013; von dem Hagen et al., 2013). We performed analyses with and without a low-pass filter for rest and all task conditions so to observe any potential changes with use of this filter (Jones et al., 2010; Nair et al., 2014). The consistency that we observed between our primary results and those obtained by this secondary task-evoked approach suggests that our results are largely robust to this processing step. However, notably, whilst all the trends persisted, some findings, like node disruption index (NDI), became non-significant and must therefore be interpreted cautiously.

The benefit of high *n* must be balanced with the genuine problem of group heterogeneity in autism research. We prioritised the reduction of group heterogeneity by stringently matching participants on age, sex and IQ at the expense of reduced *n*. This reduction of individual differences which could modulate connectivity increased the likelihood that the observed effect is real. This is, however, an exploratory study with no correction for multiple comparisons, and replication of these findings on a larger scale is necessary to validate the putative endophenotype that we report (Button et al., 2013). It is notable that previous connectivity work on “high-risk” infant siblings and adult relatives focused on mixed samples (Orekhova et al., 2014; Keehn et al., 2015) and some authors did not consider gender as a factor (Buard et al., 2013; Righi et al., 2014). Matching participants by sex is the currently recommended approach given that females with autism may not share the same neurobiological abnormalities as males (Lai et al., 2012, 2013). As such, the integrity of functional connectivity in females with ASC and their siblings remains to be elucidated, particularly in light of evidence suggesting that sex may modulate the presentation of behavioural endophenotypes of ASC (Sucksmith et al., 2013; Klusek et al., 2014).

Our analysis deals with a narrow time-window of adolescence and may not be generalisable to (male) ASC at all ages. Our findings contribute to a large pool of studies reporting hypoconnectivity in ASC. However, it is notable that a minority of studies have also reported functional and anatomical hyper-connectivity (Vissers et al., 2012; Supekar et al., 2013; Uddin et al., 2013). These findings may, in part, reflect the different ages of studied subjects (Nomi and Uddin, 2015). Findings of hyperconnectivity in ASC have been commonly associated with early life, where autistic children often exhibit brain hypertrophy (Courchesne and Pierce, 2005) which plateaus in childhood and may reverse by adolescence, with hypoconnectivity then becoming the dominant finding (Uddin et al., 2013). More recent work emphasised the developmental modulation of functional connectivity (Nomi and Uddin, 2015): their adolescent sample, like ours, showed between-network hypoconnectivity that appeared normalised in comparison to typically-developed controls in adulthood, which may explain null findings by some groups (Tyszka et al., 2014). The authors confirmed the previous suggestion of within-network hyperconnectivity in children, although hypoconnectivity between networks was also a feature of childhood ASC. In light of this, we restrict our interpretations to adolescence, and further work is needed to characterise the developmental course of functional connectivity in ASC and genetic relatives.

Movement noise is an important issue of concern in analyses of functional connectivity, as it may create artefacts of hypoconnectivity (Power et al., 2012; Satterthwaite et al., 2012; van Dijk et al., 2012). Our analysis pipeline involved regression of 32 noise variables (Satterthwaite et al., 2012), filtering and despiking. We conducted several checks which suggest that our approach adequately controlled for movement artefact. Our data speaks against an interpretation of hypoconnectivity caused by artefacts: correlations between global functional connectivity and movement parameters reflected that most participants were clustered together as “low movers” and the few who moved more (in the ASC and sibling groups) actually tended to show higher rather than lower connectivity.

Our analysis found differences in absolute but not normalised clustering coefficient (*C*) or global efficiency (*E*) in any of the four conditions. These results replicate the previous findings of Barttfeld et al. (2011, 2012), who found significantly lower non-normalised *C* during resting state and an auditory oddball task, and of Rudie et al. (2013) who found significantly lower non-normalised C in ASC during resting state, and a trend in the same direction for normalised *C* (neither group used our measure of efficiency). Together these findings suggest that changes in clustering coefficient are primarily driven by the propensity for reduction in whole-brain network connectivity, rather than by more subtle network reconfiguration. They again corroborate the fact evident from the other data in our report: despite lacking a diagnosis of autism, genetically-related but otherwise typically developing siblings of people with autism differ quantitatively from unrelated members of the public.

We finally comment on the implications and further directions arising from our use of several tasks. The robustness of the hypoconnectivity endophenotype across several functional contexts (two cognitive tasks and resting state) and the overall consistency of findings with or without low-pass filtering make the findings robust. Such robustness is also in-line with recent evidence suggesting the broad convergence and similarity of large-scale whole-brain functional connectivity maps at rest and across multiple tasks (Cole et al., 2014), and is shown in the correlations we saw between connectivity in each condition, including the Eyes task (Supplementary Materials, 6). However we cannot claim that hypoconnectivity is state-independent, given our failure to find the endophenotype effect in the Eyes task. We note, however, that endophenotypes have been seen in this task before (Holt et al., 2014) and are not contradicted by the present findings (see Supplementary Materials, 5). Future studies need to clarify the clinical utility of hypoconnectivity by searching for the specificity and positive predictive values of such endophenotypes (Rubinov and Bullmore, 2013). Given the diverse presentations of ASC and their polygenic aetiology, the identification of features specific to diagnosed individuals and their relatives on the broader autism spectrum may clarify the mechanisms underlying ASC, and the search for specific genes and targeted interventions.

## 5 Conclusion

In summary, this multitask investigation compared well-matched adolescent groups of male autistic participants, unaffected siblings and typically developing controls to show that siblings of people with autism differ significantly from typically-developing controls in neural connectivity and measures of network density. This was evident during two cognitive tasks (most particularly the Embedded Figures task) and during rest, and constitutes an endophenotype of autism in these matched adolescent participants. Our analysis revealed that brain connectivity in siblings was more similar to that of participants with ASC in presentation, which may underlie the behavioural similarities between these groups. As hypoconnectivity seems to be shared by individuals with genes conferring vulnerability for autism, it may be an endophenotype which lends weight to previous suggestions that ASCs arise from dysfunction of neural connectivity.

## Figures and Tables

**Table 1:** The demographics of each experimental group and results of F-tests between them. Means are displayed with standard deviations in parentheses (), and range in square brackets [].

*N*: 42 (14 ASC × 14 SIBS × 14 CON)				
	ASC	Siblings (SIBS)	Controls (CON)	Matching (*F*)
Age	15.05 (1.9) [6.45]	15.11 (2.0) [6.93]	15.1 (1.8) [5.33]	*F*(2, 41) = .006, p = .994.
Full-scale IQ	104.79 (14.6) [51]	112.43 (11.4) [32]	113.43 (9.1) [31]	*F*(2, 41) = 2.205, p = .124
Verbal IQ	103.5 (18.5) [64]	110 (12) [37]	110.5 (6.9) [26]	*F*(2, 41) = 1.649, p = .205
Performance IQ	106.1 (16.8) [46]	110 (11.5) [34]	113.3 (10.4) [34]	*F*(2, 41) = 1.123, p = .336
AQ (Autism-Spectrum Quotient)	39.14 (7.2) [28]	10.79 (6.3) [23]	8.86 (5.6) [21]	*F*(2, 41) = 97.882, p = .000
SRS (Social communication score)	112.9 (38.2) [138]	18.4 (15.2) [53]	14.7 (10.4) [35]	*F*(2, 41) = 72.472, p = .000

## Appendix A Supplementary data

Supplementary Material

Figure S2 The node strength of a representative participant with ASC (A), a sibling (B) and a control participant (C) are displayed along the Y axes, in each case plotted against the node strength of the ‘typical’ brain (averaged across the whole control group).

Figure S4 Strength distribution for all participants pooled in each task.

## References

[bb0087] Achard S., Delon-Martin C., Vértes P.E., Renard F., Schenck M., Schneider F., Heinrich C., Kremer S., Bullmore E.T. (2012). Hubs of brain functional networks are radically reorganized in comatose patients. Proc. Natl. Acad. Sci. U S A..

[bb00114] Assaf M., Jagannathan K., Calhoun V.D., Miller L., Stevens M.C., Sahl R., O'Boyle J.G., Schultz R.T., Pearlson G.D. (2010). Abnormal functional connectivity of default mode sub-networks in autism spectrum disorder patients. Neuroimage.

[bb0098] Baron-Cohen S., Hammer J. (1997). Parents of children with Asperger syndrome: what is the cognitive phenotype?. J Cogn Neurosci.

[bb0019] Baron-Cohen S., Ring H., Chitnis X., Wheelwright S., Gregory L., Williams S., Brammer M., Bullmore E. (2006). fMRI of parents of children with Asperger syndrome: a pilot study. Brain Cogn..

[bb006] Baron-Cohen S., Wheelwright S., Hill J., Raste Y., Plumb I. (2001). The “reading the Mind in the Eyes” test revised version: a study with normal adults, and adults with Asperger syndrome or high-functioning autism. J. Child Psychol. Psychiatry.

[bb00132] Baron-Cohen S., Wheelwright S., Skinner R., Martin J., Clubley E. (2001). The autism-Spectrum quotient (AQ): evidence from Asperger syndrome/high-functioning autism, males and females, scientists and mathematicians. J. Autism Dev. Disord..

[bb0038] Barttfeld P., Wicker B., Cukier S., Navarta S., Lew S., Leiguarda R., Sigman M. (2012). State-dependent changes of connectivity patterns and functional brain network topology in autism spectrum disorder. Neuropsychologia.

[bb0037] Barttfeld P., Wicker B., Cukier S., Navarta S., Lew S., Sigman M. (2011). A big-world network in ASD: dynamical connectivity analysis reflects a deficit in long-range connections and an excess of short-range connections. Neuropsychologia.

[bb0083] Beall E.B., Lowe M.J. (2014). SimPACE: generating simulated motion corrupted BOLD data with synthetic-navigated acquisition for the development and evaluation of SLOMOCO: a new, highly effective slicewise motion correction. Neuroimage.

[bb00123] Belmonte M.K., Allen G., Beckel-Mitchener A., Boulanger L.M., Carper R.A., Webb S.J. (2004). Autism and abnormal development of brain connectivity. J. Neurosci..

[bb00133] Belmonte M.K., Cook E.H., Anderson G.M., Rubenstein J.L.R., Greenough W.T., Beckel-Mitchener A., Courchesne E., Boulanger L.M., Powell S.B., Levitt P.R., Perry E.K., Jiang Y.H., DeLorey T.M., Tierney E. (2004). Autism as a disorder of neural information processing: directions for research and targets for therapy. Mol. Psychiatry.

[bb002] Berg J.M., Geschwind D.H. (2012). Autism genetics: searching for specificity and convergence. Genome.

[bb0091] Best C.S., Moffat V.J., Power M.J., Owens D.G., Johnstone E.C. (2008). The boundaries of the cognitive phenotype of autism: theory of mind, central coherence and ambiguous figure perception in young people with autistic traits. J. Autism Dev. Disord..

[bb0063] Bölte S., Poustka F. (2006). The broader cognitive phenotype of autism in parents: how specific is the tendency for local processing and executive dysfunction?. J. Child Psychol. Psychiatry Allied Discip..

[bb0090] Briskman J., Happé F., Frith U. (2001). Exploring the cognitive phenotype of autism: weak “central coherence” in parents and siblings of children with autism: II. Real-life skills and preferences. J. Child Psychol. Psychiatry.

[bb00128] Buard I., Rogers S.J., Hepburn S., Kronberg E., Rojas D.C. (2013). Altered oscillation patterns and connectivity during picture naming in autism. Front. Hum. Neurosci..

[bb0086] Bullmore E., Sporns O. (2012). The economy of brain network organization. Nat. Rev. Neurosci..

[bb9000] Bullmore E., Sporns O. (2009). Complex brain networks: graph theoretical analysis of structural and functional systems. Nat. Rev. Neurosci..

[bb00127] Button K.S., Ioannidis J.P., Mokrysz C., Nosek B.A., Flint J., Robinson E.S., Munafò M.R. (2013). Power failure: why small sample size undermines the reliability of neuroscience. Nat. Rev. Neurosci..

[bb0060] Cannon T.D., Keller M.C. (2006). Endophenotypes in the genetic analyses of mental disorders. Annu. Rev. Clin. Psychol..

[bb00131] Cole M.W., Bassett D.S., Power J.D., Braver T.S., Petersen S.E. (2014). Intrinsic and task-evoked network architectures of the human brain. Neuron.

[bb003] Colvert E., Tick B., McEwen F., Stewart C., Curran S.R., Woodhouse E., Gillan N., Hallett V., Lietz S., Garnett T., Ronald A., Plomin R., Rijsdijk F., Happé F., Bolton P. (2015). Heritability of autism spectrum disorder in a UK population-based twin sample. J.A.M.A. Psychiatry.

[bb0011] Constantino J.N., Zhang Y., Frazier T., Abbacchi A.M., Law P. (2010). Sibling recurrence and the genetic epidemiology of autism. Am. J. Psychiatry.

[bb00124] Courchesne E., Pierce K. (2005). Why the frontal cortex in autism might be talking only to itself: local over-connectivity but long-distance disconnection. Curr. Opin. Neurobiol..

[bb0066] Cox R.W. (1996). AFNI: software for analysis and visualization of functional magnetic resonance Neuroimages. Comput. Biomed. Res..

[bb00105] Damarla S.R., Keller T.A., Kana R.K., Cherkassky V.L., Williams D.L., Minshew N.J., Just M.A. (2010). Cortical underconnectivity coupled with preserved visuospatial cognition in autism: evidence from an fMRI study of an embedded figures task. Autism Res..

[bb0018] Dawson G., Webb S.J., Wijsman E., Schellenberg G., Estes A., Munson J., Faja S. (2005). Neurocognitive and electrophysiological evidence of altered face processing in parents of children with autism: implications for a model of abnormal development of social brain circuitry in autism. Dev. Psychopathol..

[bb0032] Di Martino A., Fair D.A., Kelly C., Satterthwaite T.D., Castellanos F.X., Thomason M.E., Craddock R.C., Luna B., Leventhal B.L., Zuo X.-N., Milham M.P. (2014). Unraveling the Miswired connectome: a developmental perspective. Neuron.

[bb0064] Ekman R. (1975). Pictures of facial affect. Emotion.

[bb00120] Fishman I., Keown C.L., Lincoln A.J., Pineda J.A., Müller R.-A. (2014). Atypical cross talk between mentalizing and mirror neuron networks in autism spectrum disorder. J.A.M.A. Psychiatry.

[bb0054] Floris D.L., Chura L.R., Holt R.J., Suckling J., Bullmore E.T., Baron-Cohen S., Spencer M.D. (2013). Psychological correlates of handedness and corpus callosum asymmetry in autism: the left hemisphere dysfunction theory revisited. J. Autism Dev. Disord..

[bb0023] Fox M.D., Raichle M.E. (2007). Spontaneous fluctuations in brain activity observed with functional magnetic resonance imaging. Nat. Rev. Neurosci..

[bb0074] Fox M.D., Snyder A.Z., Vincent J.L., Raichle M.E. (2007). Intrinsic fluctuations within cortical systems account for intertrial variability in human behavior. Neuron.

[bb0097] Gerdts J.A., Bernier R., Dawson G., Estes A. (2013). The broader autism phenotype in simplex and multiplex families. J. Autism Dev. Disord..

[bb0028] Geschwind D.H., Levitt P. (2007). Autism spectrum disorders: developmental disconnection syndromes. Curr. Opin. Neurobiol..

[bb008] Gottesman I.I., Gould T.D. (2003). The endophenotype concept in psychiatry: etymology and strategic intentions. Am. J. Psychiatry.

[bb0070] Gotts S.J., Saad Z.S., Jo H.J., Wallace G.L., Cox R.W., Martin A. (2013). The perils of global signal regression for group comparisons: a case study of autism spectrum disorders. Front. Hum. Neurosci..

[bb00108] Greicius M.D., Krasnow B., Reiss A.L., Menon V. (2003). Functional connectivity in the resting brain: a network analysis of the default mode hypothesis. Proc. Natl. Acad. Sci. U S A..

[bb005] Grønborg T.K., Schendel D.E., Parner E.T. (2013). Recurrence of autism spectrum disorders in full- and half-siblings and trends over time: a population-based cohort study. J.A.M.A. Pediatr..

[bb00106] Gusnard D.A., Raichle M.E., Raichle M.E. (2001). Reviews searching for a baseline: functional imaging and the resting human brain. Nat. Rev. Neurosci..

[bb0088] Hahamy A., Behrmann M., Malach R. (2015). The idiosyncratic brain: distortion of spontaneous connectivity patterns in autism spectrum disorder. Nat. Neurosci..

[bb0089] Happé F., Frith U. (2006). The weak coherence account: detail-focused cognitive style in autism spectrum disorders. J. Autism Dev. Disord..

[bb007] Hoekstra R.A., Bartels M., Verweij C.J., Boomsma D.I. (2007). Heritability of autistic traits in the general population. Arch. Pediatr. Adolesc. Med..

[bb0022] Holt R.J., Chura L.R., Lai M.-C., Suckling J., von dem Hagen E., Calder A.J., Bullmore E.T., Baron-Cohen S., Spencer M.D. (2014). “Reading the Mind in the Eyes”: an fMRI study of adolescents with autism and their siblings. Psychol. Med..

[bb0014] Hurley R.S., Losh M., Parlier M., Reznick J.S., Piven J. (2007). The broad autism phenotype questionnaire. J. Autism Dev. Disord..

[bb0056] Jarrold C., Brock J. (2004). To match or not to match? Methodological issues in autism-related research. J Autism Dev Disord..

[bb0084] Jenkinson M., Bannister P., Brady M., Smith S. (2002). Improved optimization for the robust and accurate linear registration and motion correction of brain images. Neuroimage.

[bb0067] Jenkinson M., Beckmann C.F., Behrens T.E., Woolrich M.W., Smith S.M. (2012). FSL. Neuroimage.

[bb0062] Jolliffe T., Baron-Cohen S. (1997). Are people with autism and Asperger syndrome faster than normal on the embedded figures test?. J. Child Psychol. Psychiatry Allied Discip..

[bb0075] Jones T.B., Bandettini P.A., Kenworthy L., Case L.K., Milleville S.C., Martin A., Birn R.M. (2010). Sources of group differences in functional connectivity: an investigation applied to autism spectrum disorder. Neuroimage.

[bb00102] Just M.A., Cherkassky V.L., Keller T.A., Kana R.K., Minshew N.J. (2007). Functional and anatomical cortical underconnectivity in autism: evidence from an fmri study of an executive function task and corpus callosum morphometry. Cereb. Cortex.

[bb0092] Just M.A., Keller T.A., Malave V.L., Kana R.K., Varma S. (2012). Autism as a neural systems disorder: a theory of frontal-posterior underconnectivity. Neurosci. Biobehav. Rev..

[bb00101] Just M.A., Newman S.D., Keller T.A., McEleney A., Carpenter P.A. (2004). Imagery in sentence comprehension: an fMRI study. Neuroimage.

[bb009] Kalueff A.V., Stewart A.M., Song C., Gottesman I.I. (2015). Targeting dynamic interplay among disordered domains or endophenotypes to understand complex neuropsychiatric disorders: translational lessons from preclinical models. Neurosci. Biobehav. Rev..

[bb00104] Kana R.K., Keller T.A., Cherkassky V.L., Minshew N.J., Just M.A. (2009). Atypical frontal-posterior synchronization of theory of Mind regions in autism during mental state attribution. Soc. Neurosci..

[bb0029] Kana R.K., Libero L.E., Moore M.S. (2011). Disrupted cortical connectivity theory as an explanatory model for autism spectrum disorders. Phys. Life Rev..

[bb0099] Keehn B., Shih P., Brenner L.A., Townsend J., Müller R.-A. (2013). Functional connectivity for an “island of sparing” in autism spectrum disorder: an fMRI study of visual search. Hum. Brain Mapp..

[bb0035] Keehn B., Vogel-Farley V., Tager-Flusberg H., Nelson C.A. (2015). Atypical hemispheric specialization for faces in infants at risk for autism spectrum disorder. Autism Res..

[bb00113] Kennedy D.P., Courchesne E. (2008). Functional abnormalities of the default network during self- and other-reflection in autism. Soc. Cogn. Affect. Neurosci..

[bb00112] Kennedy D.P., Redcay E., Courchesne E. (2006). Failing to deactivate: resting functional abnormalities in autism. Proc. Natl. Acad. Sci. U S A..

[bb00100] Keown C.L., Shih P., Nair A., Peterson N., Mulvey M.E., Müller R.A. (2013). Local functional overconnectivity in posterior brain regions is associated with symptom severity in autism spectrum disorders. Cell Rep..

[bb00130] Klusek J., Losh M., Martin G.E. (2014). Sex differences and within-family associations in the broad autism phenotype. Autism.

[bb00103] Koshino H., Carpenter P.A., Minshew N.J., Cherkassky V.L., Keller T.A., Just M.A. (2005). Functional connectivity in an fMRI working memory task in high-functioning autism. Neuroimage.

[bb0046] Lai M.C., Lombardo M.V., Ruigrok A.N., Chakrabarti B., Wheelwright S.J., Auyeung B., Allison C., Baron-Cohen S., Bolton P.F., Bullmore E.T., Carrington S., Catani M., Craig M.C., Daly E.M., Deoni S.C., Ecker C., Happé F., Henty J., Jezzard P., Johnston P., Jones D.K., Madden A., Mullins D., Murphy C.M., Murphy D.G.M., Pasco G., Sadek S.A., Spain D., Stewart R., Suckling J., Williams S.C., MRC AIMS Consortium (2012). Cognition in males and females with autism: similarities and differences. PLOS One.

[bb0047] Lai M.C., Lombardo M.V., Suckling J., Ruigrok A.N., Chakrabarti B., Ecker C., Deoni S.C., Craig M.C., Murphy D.G., Bullmore E.T., Baron-Cohen S., MRC AIMS Consortium (2013). Biological sex affects the neurobiology of autism. Brain.

[bb0058] Le Couteur A., Lord C., Rutter M. (2003). The autism diagnostic interview-revised (ADI-R). J. Cancer.

[bb0045] Lebel C., Beaulieu C. (2011). Longitudinal development of human brain wiring continues from childhood into adulthood. J. Neurosci..

[bb004] Lichtenstein P., Carlström E., Råstam M., Gillberg C., Anckarsäter H. (2010). The genetics of autism spectrum disorders and related neuropsychiatric disorders in childhood. Am. J. Psychiatry.

[bb0036] Lisiecka D.M., Holt R., Tait R., Ford M., Lai M.-C., Chura L.R., Baron-Cohen S., Spencer M.D., Suckling J. (2015). Developmental white matter microstructure in autism phenotype and corresponding endophenotype during adolescence. Transl. Psychiatry.

[bb00110] Lombardo M.V., Chakrabarti B., Bullmore E.T., Wheelwright S.J., Sadek S.A., Suckling J., Baron-Cohen S., MRC AIMS Consortium (2010). Shared neural circuits for mentalizing about the self and others. J. Cogn. Neurosci..

[bb0057] Lord C., Risi S., Lambrecht L., Cook E.H., Leventhal B.L., Dilavore P.C., Pickles A., Rutter M. (2000). The autism diagnostic observation schedule—generic: a standard measure of social and communication deficits associated with the spectrum of autism. J. Autism Dev. Disord..

[bb0010] Losh M., Adolphs R., Poe M.D., Couture S., Penn D., Baranek G.T., Piven J. (2009). Neuropsychological profile of autism and the broad autism phenotype. Arch. Gen. Psychiatry.

[bb0048] Luders E., Narr K.L., Thompson P.M., Toga A.W. (2009). Neuroanatomical correlates of intelligence. Intelligence.

[bb00118] Lynch C.J., Uddin L.Q., Supekar K., Khouzam A., Phillips J., Menon V. (2013). Default mode network in childhood autism: posteromedial cortex heterogeneity and relationship with social deficits. Biol. Psychiatry.

[bb00125] Markram K., Markram H. (2010). The intense world theory — a unifying theory of the neurobiology of autism. Front. Hum. Neurosci..

[bb00111] Mars R.B., Neubert F.-X., Noonan M.P., Sallet J., Toni I., Rushworth M.F.S. (2012). On the relationship between the “default mode network” and the “social brain.”. Front Hum Neurosci.

[bb0025] Minshew N.J., Goldstein G. (1998). Autism as a disorder of complex information processing. Ment. Retard. Dev. Disabil. Res. Rev. Review.

[bb9005] Menon V. (2011). Large-scale brain networks and psychopathology: a unifying triple network model. Trends Cogn. Sci..

[bb00121] Mostofsky S.H., Burgess M.P., Gidley Larson J.C. (2007). Increased motor cortex white matter volume predicts motor impairment in autism. Brain.

[bb0050] Mottron L. (2004). Matching strategies in cognitive research with individuals with high-functioning autism: current practices, instrument biases, and recommendations. J. Autism Dev. Disord..

[bb00126] Mueller S., Keeser D., Samson A.C., Kirsch V., Blautzik J., Grothe M., Erat O., Hegenloh M., Coates U., Reiser M.F., Hennig-Fast K., Meindl T. (2013). Convergent findings of altered functional and structural brain connectivity in individuals with high functioning autism: a multimodal MRI study. PLOS One.

[bb0039] Müller R.A., Shih P., Keehn B., Deyoe J.R., Leyden K.M., Shukla D.K. (2011). Underconnected, but how? A survey of functional connectivity MRI studies in autism spectrum disorders. Cereb. Cortex.

[bb0076] Nair A., Keown C.L., Datko M., Shih P., Keehn B., Müller R.A. (2014). Impact of methodological variables on functional connectivity findings in autism spectrum disorders. Hum. Brain Mapp..

[bb0049] Neubauer A.C., Fink A. (2009). Intelligence and neural efficiency: measures of brain activation versus measures of functional connectivity in the brain. Intelligence.

[bb9010] Nomi J.S., Uddin L.Q. (2015). Developmental changes in large-scale network connectivity in autism. NeuroImage: Clinical.

[bb0017] Nydén A., Hagberg B., Goussé V., Rastam M. (2011). A cognitive endophenotype of autism in families with multiple incidence. Res. Autism Spectr. Disord..

[bb0033] Orekhova E.V., Elsabbagh M., Jones E.J., Dawson G., Charman T., Johnson M.H., BASIS Team (2014). EEG hyper-connectivity in high-risk infants is associated with later autism. J. Neurodev. Disord..

[bb00115] Paakki J.-J., Rahko J., Long X., Moilanen I., Tervonen O., Nikkinen J., Starck T., Remes J., Hurtig T., Haapsamo H., Jussila K., Kuusikko-Gauffin S., Mattila M.-L., Zang Y., Kiviniemi V. (2010). Alterations in regional homogeneity of resting-state brain activity in autism spectrum disorders. Brain Res..

[bb0069] Patel A.X., Kundu P., Rubinov M., Jones P.S., Vértes P.E., Ersche K.D., Suckling J., Bullmore E.T. (2014). A wavelet method for modeling and despiking motion artifacts from resting-state fMRI time series. Neuroimage.

[bb0015] Pickles A., Starr E., Kazak S., Bolton P., Papanikolaou K., Bailey A., Goodman R., Rutter M. (2000). Variable expression of the autism broader phenotype: findings from extended pedigrees. J. Child Psychol. Psychiatry.

[bb0013] Piven J., Palmer P., Jacobi D., Childress D., Arndt S. (1997). Broader autism phenotype: evidence from a family history study of multiple-incidence autism families. Am. J. Psychiatry.

[bb0094] Posthuma D., De Geus E.J., Mulder E.J., Smit D.J., Boomsma D.I., Stam C.J. (2005). Genetic components of functional connectivity in the brain: the heritability of synchronization likelihood. Hum. Brain Mapp..

[bb0077] Power J.D., Barnes K.A., Snyder A.Z., Schlaggar B.L., Petersen S.E. (2012). Spurious but systematic correlations in functional connectivity MRI networks arise from subject motion. Neuroimage.

[bb0082] Power J.D., Barnes K.A., Snyder A.Z., Schlaggar B.L., Petersen S.E. (2013). Steps toward optimizing motion artifact removal in functional connectivity MRI; a reply to Carp. Neuroimage.

[bb0071] Power J.D., Cohen A.L., Nelson S.M., Wig G.S., Barnes K.A., Church J.A., Vogel A.C., Laumann T.O., Miezin F.M., Schlaggar B.L., Petersen S.E. (2011). Functional network organization of the human brain. Neuron.

[bb0078] Power J.D., Mitra A., Laumann T.O., Snyder A.Z., Schlaggar B.L., Petersen S.E. (2014). Methods to detect, characterize, and remove motion artifact in resting state fMRI. Neuroimage.

[bb0079] Power J.D., Schlaggar B.L., Petersen S.E. (2015). Recent progress and outstanding issues in motion correction in resting state fMRI. Neuroimage.

[bb00107] Raichle M.E., MacLeod A.M., Snyder A.Z., Powers W.J., Gusnard D.A., Shulman G.L. (2001). A default mode of brain function. Proc. Natl. Acad. Sci. U S A..

[bb0041] Ray S., Miller M., Karalunas S., Robertson C., Grayson D.S., Cary R.P., Hawkey E., Painter J.G., Kriz D., Fombonne E., Nigg J.T., Fair D.A. (2014). Structural and functional connectivity of the human brain in autism spectrum disorders and attention-deficit/hyperactivity disorder: a rich club-organization study. Hum. Brain Mapp..

[bb00119] Redcay E., Moran J.M., Mavros P.L., Tager-Flusberg H., Gabrieli J.D., Whitfield-Gabrieli S. (2013). Intrinsic functional network organization in high-functioning adolescents with autism spectrum disorder. Front. Hum. Neurosci..

[bb0034] Righi G., Tierney A.L., Tager-Flusberg H., Nelson C.A. (2014). Functional connectivity in the first year of life in infants at risk for autism spectrum disorder: an EEG Study. PLOS One.

[bb001] Ronald A., Hoekstra R.A. (2011). Autism spectrum disorders and autistic traits: a decade of new twin studies. Am. J. Med. Genet. B Neuropsychiatr. Genet..

[bb0065] Rorden C., Karnath H.-O., Bonilha L. (2007). Improving lesion-symptom mapping. J. Cogn. Neurosci..

[bb0052] Rubinov M., Bullmore E. (2013). Fledgling pathoconnectomics of psychiatric disorders. Trends Cogn. Sci. (Regul. Ed.).

[bb0072] Rubinov M., Knock S.A., Stam C.J., Micheloyannis S., Harris A.W., Williams L.M., Breakspear M. (2009). Small-world properties of nonlinear brain activity in schizophrenia. Hum. Brain Mapp..

[bb0051] Rubinov M., Sporns O. (2010). Complex network measures of brain connectivity: uses and interpretations. Neuroimage.

[bb9020] Rubinov M., Sporns O. (2011). Weight-conserving characterization of complex functional brain networks. Neuroimage.

[bb9025] Rudie J.D., Brown J.A., Beck-Pancer D., Hernandez L.M., Dennis E.L., Thompson P.M., Dapretto M. (2013). Altered functional and structural brain network organization in autism. NeuroImage: Clinical.

[bb0040] Rudie J.D., Brown J.A., Beck-Pancer D., Hernandez L.M., Dennis E.L., Thompson P.M., Bookheimer S.Y., Dapretto M. (2012). Altered functional and structural brain network organization in autism. Neuroimage Clin..

[bb0093a] Rutter M., Bailey A., Lord C.M. (2003). Social Communication Questionnaire.

[bb0093] Ruzich E., Allison C., Smith P., Watson P., Auyeung B., Ring H., Baron-cohen S. (2015). Measuring autistic traits in the general population: a systematic review of the autism-spectrum quotient (AQ) in a nonclinical population sample of 6,900 typical adult males and females. Mol. Autism.

[bb0068] Satterthwaite T.D., Elliott M.A., Gerraty R.T., Ruparel K., Loughead J., Calkins M.E., Eickhoff S.B., Hakonarson H., Gur R.C., Gur R.E., Wolf D.H. (2013). An improved framework for confound regression and filtering for control of motion artifact in the preprocessing of resting-state functional connectivity data. Neuroimage.

[bb0080] Satterthwaite T.D., Wolf D.H., Loughead J., Ruparel K., Elliott M.A., Hakonarson H., Gur R.C., Gur R.E. (2012). Impact of in-scanner head motion on multiple measures of functional connectivity: relevance for studies of neurodevelopment in youth. Neuroimage.

[bb00109] Schilbach L., Eickhoff S.B., Mojzisch A., Vogeley K. (2008). What's in a smile? Neural correlates of facial embodiment during social interaction. Soc. Neurosci..

[bb0096] Schutte N.M., Hansell N.K., de Geus E.J., Martin N.G., Wright M.J., Smit D.J. (2013). Heritability of resting state EEG functional connectivity patterns. Twin Res. Hum. Genet..

[bb0061] Shah A., Frith U. (1983). An islet of ability in autistic children: a research note. J. Child Psychol. Psychiatry.

[bb0095] Smit D.J., Stam C.J., Posthuma D., Boomsma D.I., De Geus E.J. (2008). Heritability of “small-world” networks in the brain: a graph theoretical analysis of resting-state EEG functional connectivity. Hum. Brain Mapp..

[bb0043] Sowell E.R., Peterson B.S., Thompson P.M., Welcome S.E., Henkenius A.L., Toga A.W. (2003). Mapping cortical change across the human life span. Nat. Neurosci..

[bb0021] Spencer M.D., Chura L.R., Holt R.J., Suckling J., Calder A.J., Bullmore E.T., Baron-cohen S. (2012). Failure to deactivate the default mode network indicates a possible endophenotype of autism. Mol Autism.

[bb0022a] Spencer M.D., Holt R.J., Chura L.R., Calder A.J., Suckling J., Bullmore E.T., Baron-Cohen S. (2012). Atypical activation during the Embedded Figures Task as a functional magnetic resonance imaging endophenotype of autism. Brain.

[bb0020] Spencer M.D., Holt R.J., Chura L.R., Suckling J., Calder A.J., Bullmore E.T., Baron-Cohen S. (2011). A novel functional brain imaging endophenotype of autism: the neural response to facial expression of emotion. Trans. Psychiatry.

[bb00129] Sucksmith E., Allison C., Baron-Cohen S., Chakrabarti B., Hoekstra R.A. (2013). Empathy and emotion recognition in people with autism, first-degree relatives, and controls. Neuropsychologia.

[bb00122] Supekar K., Uddin L.Q., Khouzam A., Phillips J., Gaillard W.D., Kenworthy L.E., Yerys B.E., Vaidya C.J., Menon V. (2013). Brain hyperconnectivity in children with autism and its links to social deficits. Cell Rep..

[bb0042] Tyszka J.M., Kennedy D.P., Paul L.K., Adolphs R. (2014). Largely typical patterns of resting-state functional connectivity in high-functioning adults with autism. Cereb. Cortex.

[bb0031] Uddin L.Q., Supekar K., Menon V. (2013). Reconceptualizing functional brain connectivity in autism from a developmental perspective. Front. Hum. Neurosci..

[bb0055] Van Casteren M., Davis M.H. (2007). Match: a program to assist in matching the. Behav. Res. Methods.

[bb0024] Van Dijk K.R., Hedden T., Venkataraman A., Evans K.C., Lazar S.W., Buckner R.L. (2010). Intrinsic functional connectivity as a tool for human connectomics: theory, properties, and optimization. J. Neurophysiol..

[bb0081] Van Dijk K.R., Sabuncu M.R., Buckner R.L. (2012). The influence of head motion on intrinsic functional connectivity MRI. Neuroimage.

[bb0073] Van Wijk B.C., Stam C.J., Daffertshofer A. (2010). Comparing brain networks of different size and connectivity density using graph theory. PLOS One.

[bb0030] Vissers M.E., Cohen M.X., Geurts H.M. (2012). Brain connectivity and high functioning autism: a promising path of research that needs refined models, methodological convergence, and stronger behavioral links. Neurosci. Biobehav. Rev..

[bb00117] Von dem Hagen E.A., Stoyanova R.S., Baron-Cohen S., Calder A.J. (2013). Reduced functional connectivity within and between “social” resting state networks in autism spectrum conditions. Soc. Cogn. Affect. Neurosci..

[bb9030] Wechsler D. (1999). Wechsler abbreviated intelligence scale.

[bb0027] Welchew D.E., Ashwin C., Berkouk K., Salvador R., Suckling J., Baron-Cohen S., Bullmore E. (2005). Functional disconnectivity of the medial temporal lobe in Asperger's syndrome. Biol. Psychiatry.

[bb00116] Weng S.-J., Wiggins J.L., Peltier S.J., Carrasco M., Risi S., Lord C., Monk C.S. (2010). Alterations of resting state functional connectivity in the default network in adolescents with autism spectrum disorders. Brain Res..

[bb0044] Westlye L.T., Walhovd K.B., Dale A.M., Bjørnerud A., Due-Tønnessen P., Engvig A., Grydeland H., Tamnes C.K., Ostby Y., Fjell A.M. (2010). Life-span changes of the human brain white matter: diffusion tensor imaging (DTI) and volumetry. Cereb. Cortex.

[bb0012] Wheelwright S., Auyeung B., Allison C., Baron-Cohen S. (2010). Defining the broader, medium and narrow autism phenotype among parents using the autism Spectrum quotient (AQ). Mol. Autism.

[bb0016] Wong D., Maybery M., Bishop D.V., Maley A., Hallmayer J. (2006). Profiles of executive function in parents and siblings of individuals with autism spectrum disorders. Genes Brain Behav..

[bb0085] Zeng L.-L., Wang D., Fox M.D., Sabuncu M., Hu D., Ge M., Buckner R.L., Liu H. (2014). Neurobiological basis of head motion in brain imaging. Proc. Natl. Acad. Sci. U S A..

