# Supplementary material for: Whole-brain functional hypoconnectivity as an endophenotype of autism in adolescents
Source: Neuroimage Clin. 2015 Aug 7;9:140–52. doi: 10.1016/j.nicl.2015.07.015 (PMC4556734; doi:10.1016/j.nicl.2015.07.015)
Supplement: Supplementary file 1 — Supplementary Material [file mmc1.doc]

***Supplementary materials, 1: Power et al. (2011) parcellation scheme.***

Table S1: brain parcellation scheme taken from Power et al. (2011), which splits the brain into 264 8mm regions of interest (ROIs), which can be subdivided into 14 functional networks.

| **Network (names taken from Power et al [2011])** | **Identity of nodes** |
| --- | --- |
| Cerebellar | Left cerebellar crucible VI (2), right cerebellar crucible VI, right cerebellar crucible V. |
| Cingulo-opercular task control | Superior frontal gyrus (3), supplementary motor cortex (3), insular cortex (3), central opercular cortex (3), anterior cingulate, anterior supramarginal gyrus. |
| Default mode | Frontal pole (9), frontal orbital cortex, medial frontal cortex, superior frontal gyrus (4), middle frontal gyrus, paracingulate gyrus (9), anterior cingulate (2), posterior cingulate (4), angular gyrus, temporal pole (3), superior temporal gyrus, anterior middle temporal gyrus, posterior middle temporal gyrus (7), left hippocampus, temporal fusiform cortex, precuneous cortex (4), lingual gyrus, superior lateral occipital cortex (6), lateral occipital cortex (3), right cerebellar crucible I. |
| Dorsal attention | Middle frontal gyrus, precentral gyrus, middle temporal gyrus (temporo-occipital part), inferior temporal gyrus, superior parietal lobule, superior lateral occipital cortex (4). |
| Fronto-parietal task control | Frontal pole (7), superior frontal gyrus, middle frontal gyrus (5), pars opercularis, paracingulate gyrus, precentral gyrus (2), posterior supramarginal gyrus (2), angular gyrus (2), superior parietal lobule (2) temporo-occipital inferior temporal gyrus, superior lateral occipital cortex. |
| Salience | Frontal pole (4), frontal orbital cortex , middle frontal gyrus, pars triangularis, insular cortex (3), paracingulate gyrus (2), anterior cingulate (3), precentral gyrus, posterior supramarginal gyrus, precuneous. |
| Subcortical | Right thalamus (2), left thalamus, left caudate, right putamen, left putamen, right pallidum, brain stem. |
| Ventral attention | Superior frontal gyrus, pars triangularis, frontal operculum cortex, posterior superior temporal gyrus, posterior middle temporal gyrus, temporo-occipital middle temporal gyrus (2), planum temporale, supramarginal gyrus. |
| Visual | Teporo-occipital fusiform cortex, occipital fusiform gyrus (4), lingual gyrus (3), cuneal cortex (4), intracalcarine cortex (5), occipital pole (3), superior lateral occipital cortex (4), inferior lateral occipital cortex (8). |
| Sensory/somatomotor hand | Precentral gyrus (13), anterior cingulate, posterior cingulate, postcentral gyrus (11), superior parietal lobule (3), precuneous cortex. |
| Sensory/somatomotor mouth | Precentral gyrus (2), insular cortex, postcentral gyrus (2). |
| Auditory | Insular cortex (2), central opercular cortex (3), postcentral gyrus, posterior superior temporal gyrus, planum temporale (4), parietal operculum cortex (2). |
| Memory retrieval | Posterior cingulate (2), precuneous cortex (3). |
| Unnamed | Frontal pole (2), frontal orbital cortex (3), frontal medial cortex, precentral gyrus, posterior middle temporal gyrus (2), posterior inferior temporal gyrus (2), anterior inferior temporal gyrus (2), inferior temporal gyrus (temporo-occipital part [2]), anterior parahippocampal gyrus, posterior parahippocampal gyrus, posterior fusiform cortex (2), anterior fusiform gyrus, temporo-occipital fusiform gyrus, precuneous cortex, occipital pole (5), right cerebellar crucible II, left cerebellar crucible I. |

***Supplementary materials, 2: node disruption index***

*Hub* disruption was initially calculated by Achard et al (2012) as a transformation which made it possible to visualise the extent to which comatose patients differed from the node strength seen in the typical population. Similarly, we here calculated the deviance (“node disruption index” [NDI]) shown by each participant from the average node strength in the typically-developed control group.

Initially, to obtain a pattern of typical node strength for this comparison, we averaged nodal strength across all control participants. The extent to which node strength is ‘disrupted from the norm’ in each participant is thus calculated by subtracting the control-averaged strength of each node from the strength that the individual shows in the corresponding node. We obtained an NDI value for each individual as the slope of this deviance against typical values, and compared the NDI values between groups. With the assumption that individuals in the control group do not deviate markedly from the control group average, their NDI should be close to 0. In contrast, the larger the difference between node strength in an individual and the node strength of the typical population, the further from 0 that individual’s NDI would be.

To illustrate the concept, we show here a plot of an individual’s node strength (axis Y) against average nodal strength in the typical brain (axis X). Total similarity between the individual and the typical brain template would result in a perfect positive correlation; in contrast, the weaker the correlation between X and Y, the greater an individual deviates from the node strength of the typical brain. This pattern is depicted here for just one ASC, one sibling and one control participant.

INSERT SUPPLEMENTARY FIGURE S2

*Figure S2. The node strength of a representative participant with ASC (A), a sibling (B) and a control participant (C) are displayed along the Y axes, in each case plotted against the node strength of the ‘typical’ brain (averaged across the whole control group).*

***Supplementary materials, 3: Further NDI analysis.***

In order to corroborate our analysis of node disruption index and to test for the homogeneity of the ASC and sibling groups, we carried out additional calculations in which we replaced the mean control nodal strength with the mean nodal strength of a) the ASC group and b) the sibling group. As such, the calculation subtracted the respective group’s mean nodal strength from the strength that each individual showed in the corresponding node. As before, the closer the resultant value is to zero, the closer that individual conforms to the average nodal strength of their group. We analysed NDI values for each task.

Unlike when NDI was computed using an average from the control group, there were no significant group differences in NDI values for any of the four conditions, either when siblings or ASC were used to compute average nodal strength.

*Table S3A: Average NDI values for each group in each task (standard deviations in brackets) when average nodal strength of the ASC or sibling group was used for comparison.*

| NDI calculated with ASC group mean nodal strength | | | | |
| --- | --- | --- | --- | --- |
|  | Figures task | Ekman task | Eyes task | Rest |
| ASC | -.022 (.141) | -.032 (.140) | -.050 (.171) | -.007 (.074) |
| SIBS | -.088 (.156) | -.055 (.175) | -.077 (.129) | -.045 (.119) |
| CON | -.069 (.128) | -.039 (.147) | -.074 (.123) | .002 (.062) |
| NDI calculated with SIBLING group mean nodal strength | | | | |
|  | Figures task | Ekman task | Eyes task | Rest |
| ASC | -.124 (.116) | -.167 (.156) | -.100 (.165) | -.108 (.083) |
| SIBS | -.058 (.193) | -.071 (.245) | -.047 (.143) | -.032 (.158) |
| CON | -.059 (.150) | -.084 (.186) | -.076 (.129) | -.044 (.082) |

We may compare this with mean NDI values computed using average nodal strength of the control group, which were significantly different in every task but the Eyes task, in which they were marginally non-significant (see Figure 4, main manuscript):

*Table S3B: Average NDI values for each group in each task (standard deviations in brackets) when average nodal strength of the control group was used for comparison.*

| NDI calculated with control group mean nodal strength | | | | |
| --- | --- | --- | --- | --- |
|  | Figures task | Ekman task | Eyes task | Rest |
| ASC | -.192 (.127) | -.252 (.146) | -.153 (.151) | -.153 (.107) |
| SIBS | -.152 (.196) | -.184 (.211) | -.128 (.131) | -.134 (.146) |
| CON | .007 (.183) | -.039 (.215) | -.032 (.152) | -.013 (.107) |

Bearing in mind that values closer to zero reflect greatest conformance to the average strength of whichever group, it is clear that when average nodal strength is computed from the control group, individual members of the control group have scores which are on average closer to zero than do individuals from the sibling and ASC group – this occurs in every task and is quite a large difference. In comparison, when NDI is calculated from the average nodal strength of the ASC group, individuals with ASC are statistically no closer to zero (no more similar to the mean ASC nodal strength) than are individuals from the control or the sibling group (although there is a very non-significant trend in this direction in all tasks but rest). When NDI is calculated from average sibling nodal strength, individual siblings do appear to be slightly more similar to their group’s average nodal strength but not significantly more so than autistic or control individuals.

We interpret this to suggest that heterogeneity of nodal strength is greater in the sibling and ASC group, given that individual members of the ASC and sibling group are no more likely to be more similar to their group’s average nodal strength than are members of the other groups. This is not true of control participants, who are significantly more likely than ASC or sibling participants to be closer to their group’s average nodal strength in every task.

***Supplementary materials, 4: confirmation of local differences in hub topography***

Our initial analysis of hub topography involved identifying the 52 (20% of 258) nodes (henceforth ‘hubs’) with greatest strength.

Genuine differences in one network, by this method, could introduce artificial differences: if a group, for example, possesses a larger proportion of the 52 hubs in one network, by necessity they possess fewer in others. As a further corroboration of local results, therefore, we confirmed these findings with two methods. Firstly, we altered our methods of hub selection: following perusal of strength distribution for all participants (see below), all nodes with a strength of 70 or higher were defined as hubs for each participant

INSERT SUPPLEMENTARY FIGURE S4

*Figure S4: Strength distribution for all participants pooled in each task.*

A higher proportion of hubs (high-strength nodes) in one network suggests greater connectivity in that network, so we secondly corroborated our results by examining interconnectivity between that network and the remaining nodes of the parcellation scheme, and, thirdly, intraconnectivity of hubs within that network.

***Supplementary Materials, 5: Analysis and further discussion of the ‘Reading the Mind in the Eyes’ task.***

The interleaved block-design of the ‘Reading the Eyes’ did not allow for a separation of the separate conditions (i.e. mental state vs. gender judgements), as the different blocks were too short to reliably compute resting state functional connectivity. Collapsing the two conditions in this case negates the mentalising aspect of the task and makes it a non-specific task of ‘active cognitive processing’; unlike the other tasks, which specifically target visual search and processing (Figures) and emotion processing (Ekman), we cannot draw any inferences about mentalising. For brevity of the manuscript, we therefore include the remainder of the analysis of this task here in Supplementary Materials.

There was no significant group difference (i.e. no endophenotype effect) in an ANOVA comparing whole-brain connection weights in this task. In analysis of node disruption index, there was a marginally non-significant effect of group (*F* [2, 41] = 2.716, p = .079): control participants conformed most closely to the ‘typical’ nodal strength, but the difference was not as strong as in the other tasks and became weaker still in the re-analysed task-evoked approach (p = .358).

No significant differences in clustering coefficient, global efficiency, or hub topography, were seen in the original analysis or the re-analysed task-evoked approach.

A previous study examining brain activity evoked by this task in an unmatched superset of the present sample of participants (Holt et al, 2014) reported an endophenotype of reduced activation in frontotemporal social cognition regions during mentalising as compared to gender-judgement conditions (which would indeed speak against task-independence of this type of abnormality, at least). This was most striking in females but evident at trend level in males. The lack of an endophenotype in our Eyes analysis does not contradict the previous findings from this same sample, given that our methodology differed substantially and our male sample was smaller though stringently matched. In addition, Holt et al (2014) document an endophenotype effect specific to *mentalising,* a process in which both people with ASC *and* their relatives show abnormalities. We may have failed to replicate this specific endophenotype effect as we were unable to analyse the ‘Reading the Mind in the Eyes’ task in its true mentalising context.

***Supplementary materials, 6: Correlations between conditions***

We correlated the full matrices of correlations for all pairs of tasks, and rest.

*Table S6:* Correlation coefficients between functional connectivity in each task condition and at rest.

|  | Figures task | Ekman task | Resting state | Eyes task |
| --- | --- | --- | --- | --- |
| Figures task |  |  |  |  |
| Ekman task | r = .616, p = .000 |  |  |  |
| Resting state | r = .593, p = .000 | r = .452, p = .003 |  |  |
| Eyes task | r = 5.31, p = .000 | r = .571, p = .000 | r = .414, p = .006 |  |

***Supplementary materials, 7: Non-normalised graph metrics***

Prior to normalisation against 100 random networks, significant differences were observed between groups in both clustering coefficient (*C*) and global efficiency (*E*) in two of the four conditions.

In the Figures task, two clear endophenotype effects occurred with lowest *C* in the autistic group and highest *C* in controls (*F* (2, 41) = 5.102, p = .011) and a significant pattern in the same direction with *E* (*F* [2, 41] = 3.875, p = .029). The contrast of siblings and control participants was marginally non-significant in both *C* (*t* [26] = 1.993, p = 057) and *E* (*t* [26] = 1.782, p = .086), but the contrast of autistics vs. controls was highly significant in both *C* (*t* [26] = 3.789, p = .001) and *E* (*t* [26] = 3.010, p = .006).

During rest, the same significant trend was seen: lowest *C* (*F* = [2, 41] = 4.495, p = .018) and *E* (*F* [2, 41] = 3.638, p = .036) in ASC, highest in controls. Siblings occupied the intermediate position between the two groups in both measures, although direct contrasts between siblings and controls were marginally non-significant in *C* (*t* [26] = 1.818, p = .081) and *E* (*t* [26] = 1.821, p = .080). Autistic and controls did however differ statistically in both *C* (*t* [26] = 3.372, p = .002) and *E* (*t* [26] = 2.983, p = .006).

ANOVA showed no significant effects in non-normalised *C* or *E* in the Ekman task. The contrast of siblings and controls was marginally non-significant in *E* (*t* [26] = 1.893, p = .070), but the contrast of ASC and controls was significant for *C* (*t* [26] = .042) and *E* (*t* [26] = 2.367, p = .026.

In the task-evoked approach to the data (re-analysed without low-pass filter), significant differences in non-normalised *C* (*F* [2, 41] = 3.755, p = .032) and *E* (*F* [2, 41] = 3.987, p = .027) were seen in the Figures task. Direct contrasts between autistic and control participants mirrored the results above, with significant differences in *C* (*t* [26] = 2.863, p = .008) and *E* (*t* [26] = 2.797, p = .010). T-tests between controls and siblings showed differences that were borderline significant in *C* (*t* [26] = 1.978, p = .059) and *E* (*t* [26] = 2.070, p = .049). As before, all results fell below significance when normalised.

During rest, non-normalised *C* (*F* [2, 41] = 3.918, p = .028) was significantly different between groups, and the significant difference in *E* seen above dropped just marginally below significance (*F* [2, 41] = 2.723, p = .078). T-tests also mirrored the findings in the original analysis: between controls and participants, the same significant difference was seen in *C* (*t* [26] = 2.154, p = .041), though this fell below significance for *E* (*t* [26] = 1.911, p = .067). Contrasts between controls and siblings actually became significant in this re-analysed dataset, for both *C* (*t* [26] = 2.581, p = .016) and *E* (*t* [26] = 2.168, p = .039). However, all became non-significant when normalised.

***Supplementary materials, 8: corroboration of local network abnormalities, Figures Task***

A group difference in the number of DMN hubs was marginally non-significant in the main text, but this became highly significant when we examined the interregional connections between the DMN and the rest of the brain (*F* [2, 41] = 13.755, p = .000). Intermediate between controls and ASC participants, siblings showed significantly weaker connection weights than controls (*t* [26] = 3.072, = .005), who showed significantly stronger connections than ASC participants (*t* [26] = 5.795, p = .002). The endophenotype group effect seen in the number of hubs in the subcortical system (main text) was also mirrored in connection weights between the subcortical system and the rest of the brain (*F* [2, 41] = 3.297, p = .048); siblings were still intermediate but did not differ significantly from controls when compared directly (p = .093), though participants with ASC did show significantly weaker connection strengths than controls (*t* [26] = 2.536, p = .018). Differences in connection weights did not reach significance in the other networks reported above for the Figures task. The visual system showed a non-significant trend for strongest interregional connectivity in the ASC group, intermediate connectivity in siblings and weakest connectivity in controls. We note that our statistical test of interregional connectivity examined connections between the 31 visual nodes and all remaining 227 brain nodes: hypoconnectivity between visual network and other brain regions may be more specific to certain networks.

Differences in intraconnectivity (i.e. strength of correlations between nodes of a network) did not reach significance in the subcortical system or the visual system. However, the endophenotype effect reported in the main text was strongly corroborated in the intraconnectivity of the DMN (*F* [2, 41] = 7.964, p = .001). T-tests showed that control participants showed significantly greater DMN intraconnectivity than participants with ASC (*t* [26] = 4.495, p = .000) and sibling participants (*t* [26] = 2.696, p = .012) who, as in interregional connectivity, were intermediate between ASC and controls.

***Supplementary materials, 9: corroboration of local network abnormalities, Ekman Task***

Of the networks where hub topography differed between groups in the Ekman task, the DMN was the only network where endophenotype effects were confirmed by differences in interregional connection weights (*F* [2, 41] = 15.099, p = .000). Control participants showed significantly stronger connection weights than siblings (*t* [26] = 3.918, p = .001) and people with ASC (*t* [26] = 4.002, p = .000). Within network connectivity (intraconnectivity) between DMN nodes also confirmed the endophenotype reported in the main text with a group difference (*F* [2, 41] = 11.665, p = .000) and t-tests: again, autistic participants (*t* [26] = 4.002, p = .000) and siblings (*t* [26] = 4.363, p = .000) showed significantly lower DMN intraconnectivity than controls.

***Supplementary materials, 10: comparison of results with and without low-pass filter.***

To check the robustness of our findings, we re-analysed our data without a low-pass filter, an approach commonly adopted in task-evoked functional connectivity approaches which include spontaneous brain oscillations (as in rest) and task-based fluctuations (Fox et al. 2007; Van Dijk et al. 2010). Our results remained remarkably consistent between the two analyses. Where some results became marginally non-significant, their trends remained. A full list of the results of the first (low-pass filter) and second preprocessing (no low-pass filter) are presented below. Blue results in the third column show where results changed from significant to non-significant, whereas red results show where results became significant; these changes are outlined in the main text.

*Table S10:* *Comparison of main results with and without a low-pass filter.*

|  | Low-pass filter | No low-pass filter |
| --- | --- | --- |
| Global differences in functional connectivity  *t*-tests: controls x ASC  *t*-tests: controls x siblings | Group effect across four conditions: *F* (1, 39) = 4.082, p = .025.  Group effect in Figures task: *F* (2, 41) = 4.003, p = .026.  Group effect in resting state: *F* (2, 41) = 4.221, p = .022  Figures task: *t* (26) = 3.291, p = .003.  Ekman task: *t* (26) = 2.270, p = .032.  Resting state: *t* (26) = 3.427, p = .002.  No significant differences. | Group effect across four conditions: *F* (1, 39) = 4.320, p = .020  Group effect in Figures task: *F* (2, 41) = 3.741, p = .033.  Group effect in resting state: *F* (2, 41) = 3.240, p = .050  Figures task: *t* (26) = 2.808, p = .009.  Ekman task: *t* (26) = 1.790, p = .085.  Resting state: *t* (26) = 2.229, p = .035.  Rest: *t* (26) = 2.257, p = .033. |
| Clustering coefficient and global efficiency | Non-significant when normalised (see Item 7). | Non-significant when normalised (see Item 7). |
| Node disruption index  t-tests: controls x ASC  t-tests: controls x siblings | Group effect in Figures task: *F* (2, 41) = 5,322, p = .009.  Group effect in Ekman task: *F* (2, 41) = 4.422, p = .019.  Group effect in resting state: *F* (2, 41) = 2.226, p = .008  Figures task: *t* (26) = 3.354, p = .002.  Ekman task: *t* (26) = 3.061, p = .005.  Resting state: *t* (26) = 3.458, p = .002.  Figures task: *t* (26) = = 2.226, p = .035.  Resting state: *t* (26) = 2.491, p = .019. | Group effect in Figures task: *F* (2, 41) = 2.496, p = .095.  Group effect in Ekman task: *F* (2, 41) = 2.493, p = .096.  Group effect in resting state: *F* (2, 41) = 3.557, p = .038  Figures task: *t* (26) = 1.645, p = .112.  Ekman task: *t* (26) = 1.910, p = .067.  Resting state: *t* (26) = 2.189, p = .038.  Figures task: *t* (26) = 1.975, p = .059.  Figures task: *t* (26) = 2.450, p = .021. |
| Local group differences: Figures task  t-tests: controls x ASC  t-tests: controls x siblings | ASC > siblings > controls: cerebellar network (*F* [2, 41] = 8.048, p = .001), visual network (*F* [2, 41] = 4.379, p = .019).  ASC < siblings < controls: subcortical network (*F* [2, 41] = 3.672, p = .035), default mode network (DMN) (*F* [2, 41] = 3.095, p = .057).  ASC < controls < siblings: ventral attention network (*F* [2, 41 = 5.594, p = .007).  ASC > controls > siblings: cingulo-opercular task control network (*F* [2, 41] = 3.450, p = .042).  Cerebellar network: *t* (26) = 3.432, p = .002.  Visual network: *t* (26) = 2.931, p = .007.  Subcortical network: *t* (26) = 2.895, p = .008.  Default mode network: *t* (26) = 2.721, p = .011.  Visual network: *t* (26) = 2.421, p = .023. | ASC > siblings > controls: cerebellar network (*F* [2, 41] = 5.609, p = .007), visual network (*F* [2, 41] = 4.518, p = .017)  ASC < siblings < controls: subcortical network (*F* [2, 41] = 1.966, p = .154]), default mode network (*F* [2, 41] = 4.207, p = .022).  ASC < controls < siblings: ventral attention network (*F* [2, 41] = 4.018, p = .026).  ASC > controls > siblings: cingulo-opercular task control network (*F* [2, 41] = 1.168, p = .322.  Cerebellar network: *t* (26) = 3.160, p = .004.  Visual network: *t* (26) = 3.088, p = .005.  Subcortical network: *t* (26) = 1.963, p = .060.  Default mode network: *t* (26) = 2.971, p = .006.  Visual network: *t* (26) = 1.960, p = .061. |
| Local group differences: Ekman task  t-tests: controls x ASC  t-tests: controls x siblings | ASC > siblings > controls: dorsal attention network (*F* [2, 41] = 5.746, p = .006), visual network (*F* [2, 41] = 5.530, p = .008).  ASC < siblings < controls: default mode network (*F* [2, 41] = 6.381, p = .004).  Siblings > ASC > controls: cingulo-opercular task control network (*F* [2, 41] = 3.522, p = .039).  Default mode network: *t* (26) = 3.441, p = .002.  Dorsal attention network: *t* (26) = 3.121, p = .004.  Default mode network: *t* (26) = 2.796, p = .010.  Dorsal attention network: *t* (26) = 2.823, p = .009. | ASC > siblings > controls: dorsal attention network (*F* [2, 41] = 4.328, p = .020), visual network (*F* [2, 41] = 3.680, p = .034).  ASC < siblings < controls: default mode network (*F* [2, 41] = 7.826, p = .001).  Siblings > ASC > controls: cingulo-opercular task control network (*F* [2, 41] = 3.801, p = .031).  Default mode network: *t* (26) = 3.926, p = .001.  Dorsal attention network: *t* (26) = 2.822, p = .009.  Default mode network: *t* (26) = 2.895, p = .008.  Dorsal attention network: *t* (26) = 2.490, p = .019. |
