# Supplementary material for: Whole-brain functional hypoconnectivity as an endophenotype of autism in adolescents
Source: Neuroimage Clin. 2015 Aug 7;9:140–52. doi: 10.1016/j.nicl.2015.07.015 (PMC4556734; doi:10.1016/j.nicl.2015.07.015)
Supplement: Figure S2 — The node strength of a representative participant with ASC (A), a sibling (B) and a control participant (C) are displayed along the Y axes, in each case plotted against the node strength of the ‘typical’ brain (averaged across the whole control group). [file mmc2.pdf]

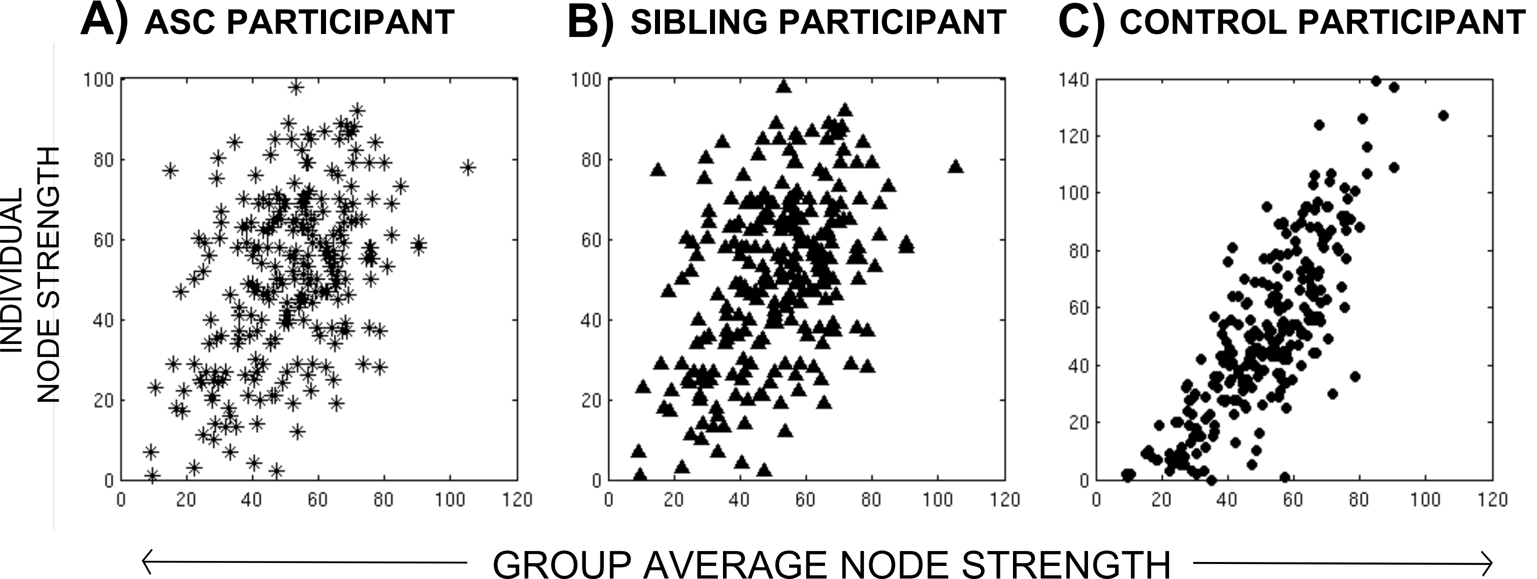
A) ASC PARTICIPANT
B) SIBLING PARTICIPANT
C) CONTROL PARTICIPANT
INDIVIDUAL NODE STRENGTH
GROUP AVERAGE NODE STRENGTH
0
20
40
60
80
100
120
140
