# Supplementary figures and images for: Whole-brain functional hypoconnectivity as an endophenotype of autism in adolescents
Source: Neuroimage Clin. 2015 Aug 7;9:140–52. doi: 10.1016/j.nicl.2015.07.015 (PMC4556734; doi:10.1016/j.nicl.2015.07.015)

FIGURES TASK

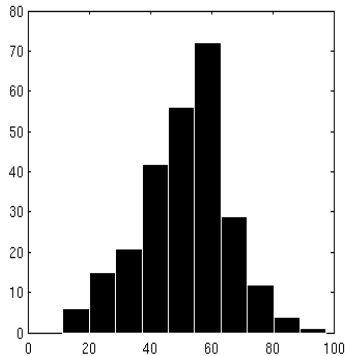

EKMAN TASK

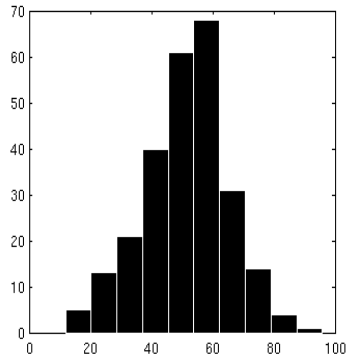

REST

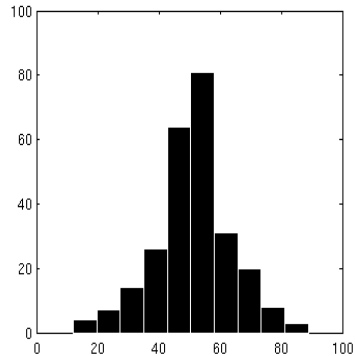

EYES TASK

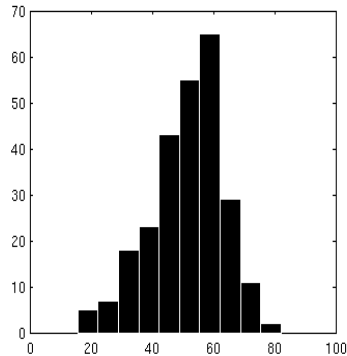

NODE STRENGTH DISTRIBUTION OF ALL PARTICIPANTS (N=42)

Supplement: Figure S4 — Strength distribution for all participants pooled in each task. [file mmc3.pdf]
